# Pellino1 deficiency reprograms cardiomyocytes energy metabolism in lipopolysaccharide-induced myocardial dysfunction

**DOI:** 10.1007/s00726-021-02978-w

**Published:** 2021-04-22

**Authors:** Chuanxi Yang, Kun Zhao, Xufeng Chen, Lei Jiang, Peng Li, Peipei Huang

**Affiliations:** 1 Department of Cardiology, Medical School of Southeast University, Nanjing, China; 2 Department of Cardiology, The First Affiliated Hospital of Nanjing Medical University, 300 Guangzhou Road, Nanjing, 210029 China; 3 Department of Emergency, The First Affiliated Hospital of Nanjing Medical University, 300 Guangzhou Road, Nanjing, 210029 China; 4 Department of Cardiology, Yangpu Hospital, Tongji University School of Medicine, Shanghai, 200090 China

**Keywords:** Pellino1, Lipopolysaccharide, Cardiomyocytes, Metabolomics, Fatty acid, Citrate cycle

## Abstract

Pellino1 has been shown to regulate proinflammatory genes by activating the nuclear factor kappa B (NF-κB) and Toll-like receptor (TLR) signaling pathways, which are important in the pathological development of lipopolysaccharide (LPS)-induced myocarditis. However, it is still unknown whether silencing Pellino1 (si-Pellino1) has a therapeutic effect on this disease. Here, we showed that silencing Pellino1 can be a potential protective strategy for abnormal myocardial energy metabolism in LPS-induced myocarditis. We used liquid chromatography electrospray–ionization tandem mass spectrometry (LC–MS/MS) to analyze samples from si-Pellino1 neonatal rat cardiac myocytes (NRCMs) treated with LPS or left untreated. After normalization of the data, metabolite interaction analysis of matched KEGG pathway associations following si-Pellino1 treatment was applied, accompanied by interaction analysis of gene and metabolite associations after this treatment. Moreover, we used western blot (WB) and polymerase chain reaction (PCR) analyses to determine the expression of genes involved in regulating cardiac energy and energy metabolism in different groups. LC–MS-based metabolic profiling analysis demonstrated that si-Pellino1 treatment could alleviate or even reverse LPS-induced cellular damage by altering cardiomyocytes energy metabolism accompanied by changes in key genes (Cs, Cpt2, and Acadm) and metabolites (3-oxoocotanoyl-CoA, hydroxypyruvic acid, lauroyl-CoA, and NADPH) in NRCMs. Overall, our study unveiled the promising cardioprotective effect of silencing Pellino1 in LPS-induced myocarditis through fuel and energy metabolic regulation, which can also serve as biomarkers for this disease.

**Supplementary Information:**

The online version contains supplementary material available at 10.1007/s00726-021-02978-w.

## Introduction

Sepsis is known to diminish oxidative metabolism in the heart and other tissues (Rudiger and Singer 2007). Myocarditis, caused mainly by bacterial or viral infections and autoimmune diseases (Hekimian and Combes 2017), is a multietiological pathological immune process involving myocardial inflammation with various clinical symptoms and outcomes, such as acute heart failure, chronic dilated cardiomyopathy, and even sudden cardiac death (Bracamonte-Baran and Cihakova 2017; Zhang et al. 2013; Sagar et al. 2012).

Notably, the occurrence and development of myocarditis involve various pathophysiological mechanisms of inflammation. The activation of multiple inflammatory regulators and related transcription factors can trigger the excessive inflammatory response, which is essential for the malignant development of cardiomyopathy (Maier et al. 2012; Akira et al. 2006). The inflammatory response and left-ventricular dysfunction induced by lipopolysaccharide (LPS) are mediated, in large part, by the host molecule Toll-like receptor 4 (TLR4) (Nemoto et al. 2002). TLR4 can trigger members of the nuclear factor kappa B (NF-κB) family and other transcription factors involved in the immune response and different cardiovascular diseases, such as ischemia–reperfusion injury and cardiac hypertrophy (Oyama et al. 2004; Timmers et al. 2008; Ha et al. 2005). Previous studies have reported variable effects of LPS on cardiac fuel and energy metabolism, suggesting that targeting inflammatory-metabolic “crosstalk” could improve outcomes in this syndrome.

The Pellino family interacts with Pelle/IRAK and has been identified to mediate the transcriptional regulation of proinflammatory genes in innate immunity through ubiquitination and then activate TLR and/or T-cell receptor (TCR) signaling (Grosshans et al. 1999b; Butler et al. 2007; Moynagh 2014). Pellino1, in particular, has been shown to play a dispensable role in activating the NF-κB family or other signal transduction pathways, which is important in T-cell activation and differentiation by the MyD88/TRIF-dependent TLR pathway (Chang et al. 2009; Kawai and Akira 2007; Choi et al. 2006; Vallabhapurapu and Karin 2009). A previous report has shown that the loss of Pellino1 can attenuate the activation of the TRIF-dependent NF-kB family due to the hyperactivation and nuclear accumulation of c-Rel in response to T-cell receptor-CD28 (TCR-CD28) signaling (Chang et al. 2011; Smith et al. 2011). In this study, we further explored whether silencing Pellino1 has therapeutic effects on LPS-induced myocarditis through multiple known or unknown regulatory mechanisms.

Currently, the immune response is the best-known underlying pathogenic mechanism and therapeutic target of bacterial myocarditis (Zhou and Yu 2017). However, the therapeutic effect is limited, and more strategies are needed to complement or replace this treatment (Kindermann et al. 2012). Since the close relationship between metabolism and signaling programs of immune cells may show a critical physiological regulation in normal or disease conditions by maintaining nutrient uptake and metabolic homeostasis (Andrejeva and Rathmell 2017), we used metabolomics to explore a new possible mechanism in the treatment of bacterial myocarditis.

In our study, we attempted to use LC–MS/MS to analyze the differences in metabolic compositions that could be vital in the occurrence and progression of LPS-induced myocarditis between samples exposed under different conditions. Thus, we could further investigate the therapeutic effect of silencing Pellino1 through metabolic mechanisms on LPS-induced myocarditis.

## Materials and methods

### Cell culture

NRCMs were prepared from 1-day-old Sprague–Dawley rats (Charles River) as previously described (Paradis et al. 2015). The cells were subsequently treated with 10 µg/mL LPS or PBS for 12 h. Adenovirus silencing Pellino1 (si-Pellino1, 2 × 107 pfu/mL) or adenoviral GFP was purchased from Shanghai GeneChem Co., Ltd. NRCMs were infected with adenovirus expressing GFP (Ad-GFP) or silencing Pellino1 (si-Pellino1) on day 1 after plating and on day 2 were treated with 10 µg/mL LPS or PBS for 12 h, after which mRNA was isolated for RT-PCR. The cells were lysed to obtain protein for western blot analysis.

### Western blot analysis

NRCMs were lysed in RIPA buffer (P0013C, Beyotime) supplemented with 1 mM PMSF (ST505, Beyotime). Thirty micrograms of total protein were subjected to electrophoresis on 10% SDS-PAGE gels (Beyotime Biotechnology, China) and transferred to polyvinylidene fluoride (PVDF) (Merck-Millipore, Shanghai, China) membranes. After the samples were blocked with 5% milk powder in TBS-Tween for 1 h, the proteins were probed with primary antibody at 4 °C overnight. After the membranes were washed three times with TBS-Tween, they were incubated for 1 h with the corresponding secondary antibody conjugated to horseradish peroxidase and then subjected to enhanced chemiluminescence (ECL) for detection of protein bands. The primary antibodies used in this study were as follows: anti-CC3 (1:1000 dilution, #9664, Cell Signaling Technology), anti-C3 (1:1000 dilution, #9662, Cell Signaling Technology), anti-Bcl2 (1:1000 dilution, #3498, Cell Signaling Technology), anti-BAX (1:1000 dilution, #5023, Cell Signaling Technology), and anti-Pellino1 (1:200 dilution, sc-271065, Santa Cruz Biotechnology), and β-Actin (1:1000 dilution, #4967, Cell Signaling Technology) was used as a loading control (*n* = 3).

### RNA analysis and real-time quantitative PCR (qRT-PCR)

Total cellular RNA isolation from NRCMs was performed using the RNazol B method and a Qiagen RNeasy kit, according to the manufacturer’s instructions. RNA was reverse transcribed (Applied Biosystems) using random hexamer priming. Real-time qRT-PCR was performed using SYBR Green reagent (Applied Biosystems) and rat-specific primers (Table S1) on the ABI Prism 7500 Sequence Detection system. GAPDH was used as an internal control. The relative gene expression levels were calculated using the 2 − △△Ct method (*n* = 3).

### Flow cytometry

We incubated all collected viable and dead cells with propidium iodide (PI) and Annexin-V (Fcmacs Biotech Co., China) in the provided binding buffer at room temperature for 30 min in the dark, according to the manufacturer’s recommendations. Then, the flow cytometry method was applied to determine the relative cell apoptosis ratio (*n* = 3).

### Metabolite extraction

Cultured NRCMs were quickly washed twice with ice-cold phosphate-buffered saline (PBS) in a cold room to remove medium components and then quickly rinsed with Milli-Q water. After the removal of water, 400 μL of 80% CH3OH/Milli-Q water was added to each culture plate. The cells were placed in liquid N_2_ for 10 min, thawed, and sonicated for 10 min. This process was repeated three times to completely extract the metabolites from the cells. Combined samples from the extraction process were then centrifuged at 10,000*g* for 5 min to pellet insoluble debris at 4 °C, with the supernatant transferred to microcentrifuge tubes and dried with a speed vacuum. Then, each dried sample was resuspended, vortexed, centrifuged, and transferred to autosampler vials for LC–MS analysis. Each group had six biological replicates.

### LC–MS and data analysis

Separation of metabolites was performed by reversed-phase LC (HP1100, Agilent Technologies, Santa Clara, CA, USA) using a reversed-phase XBridge C18 column (1.7 μm particle size, 1 × 150 mm, Waters, Milford, MA, USA), and 8 μL of each sample was injected on the C18 column for analysis. Mobile phase A consisted of H_2_O/0.1% formic acid, and mobile phase B consisted of ACN with 0.1% formic acid with a program: 1% B at 0–1 min, 15% B at 3 min, 70% B at 5 min, 85% at 9 min, 100% at 10–12 min, and subsequently return to the initial conditions with 2 min for equilibration. Furthermore, the MS program was conducted on a 6545 Quadrupole-Time of Flight system (all devices from Agilent Technologies, Santa Clare, CA, United States) as follows: both positive- and negative-ion modes with drying gas 300℃ flow 6 L/min, sheath gas 340℃ flow 11 L/min, nebulizer gas 35 psig, capillary voltage 4000 V, and fragmental voltage 135 V. The data collection (MS 100–3200 *m*/*z*, MS/MS 30–3200 *m*/*z*) was acquired by both centroid and profile stored in autoMSMS scan mode with reference masses at *m*/*z* 112.05087 and 922.009798 were set as online accurate mass calibration.

MassHunter Workstation software (version B.07.00; Agilent Technologies) was used to export mzdata format from the acquired MS data (.d). Data analysis was performed by the XCMS Online at https://xcmsonline.scripps.edu/ using three steps: data upload, parameter selection, and result interpretation. A list of the intensities of all the peaks detected was generated using the retention time and the mass-to-ratio data pairs as the parameters for each ion. MS/MS spectra of the selected putative identifications were retrieved and matched with entries in the Metlin, Massbank, the Human Metabolome Database (HMDB). The metabolomics data resulting directly from XCMS Online were used to generate the cloud plot. The binning data were normalized to the total area. PCA, partial least-squares discriminant analysis (PLS-DA), orthogonal partial least-squares discriminant analysis (OPLS-DA), and metabolites and metabolic set enrichment analysis were performed with the web-based software MetaboAnalyst 3.0 (http://www.metaboanalyst.ca).

### Statistical analyses

Data was presented as mean ± standard error of the mean (SEM). Using GraphPad Prism 4.0 (GraphPad software Inc., CA, USA), statistical significance among multiple groups was evaluated by one-way analysis of variance (ANOVA) with the Bonferroni post hoc test. A two-tailed *P* value < 0.05 was considered statistically significant.

## Results

### Silencing Pellino1 alleviates LPS-induced cardiomyocyte apoptosis and inflammation

**Fig. 1:**
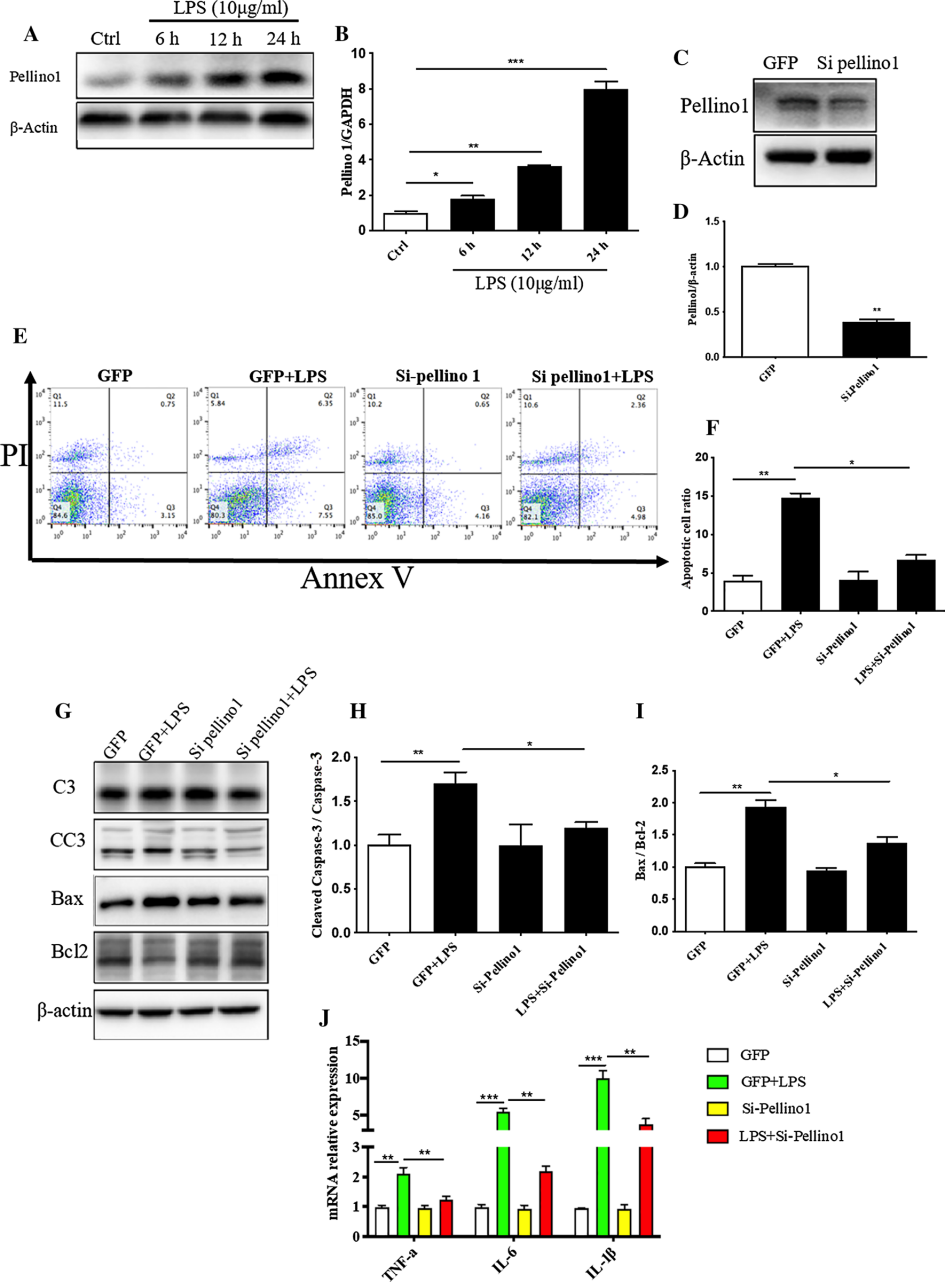
Silencing Pellino1 alleviates LPS-induced cardiomyocyte apoptosis and inflammation in vitro. **a**, **b** Representative western blot of the expression of Pellino1 in the LPS-induced cardiomyocytes. The bar graph shows that the expression of Pellino1 increased gradually with LPS treatment time. β-actin was detected as the loading control. **c**, **d** The efficacy of silencing Pellino1 via an adenovirus was assessed by western blotting. β-actin was detected as the loading control. **e**, **f** Apoptosis of cardiomyocytes exposed to LPS with or without the Pellino1-silencing adenovirus. The right bar graph shows the results of statistical analysis of the apoptosis ratios. **g**–**i** Representative western blot of the expression of Bax, Bcl2, CC3, and C3 in LPS-induced cardiomyocytes with/without si-Pellino1 treatment. The relative protein levels were normalized to the β-actin levels. The data are shown as the mean ± standard error of the mean of triplicates and are representative of three independent experiments performed. **j** Representative qRT-PCR of mRNA expression of TNF-α, IL-1β, and IL-6 in the LPS-induced cardiomyocytes with/without adenovirus silencing Pellino1. All mRNA expression was normalized to that of GAPDH. *n* = 6, results are expressed as the mean ± SD, *P < 0.05, **P < 0.01, ***P < 0.001, compared with the ctrl or GFP group

To verify the role of Pellino1 in LPS-induced septic cardiomyopathy, we first examined its expression in cardiomyocytes after LPS treatment. Western blot analysis showed that the expression of Pellino1 increased gradually with LPS treatment time (Fig. 1a, b). To further investigate whether silencing Pellino1 can ameliorate LPS-induced apoptosis, we used adenovirus inhibition of Pellino1 before LPS treatment, significantly reducing the Pellino1 expression levels in NRCMs (Fig. 1c, d). Flow cytometry showed that the LPS-induced increase in apoptosis was partially reversed by silencing Pellino1 (Fig. 1e, f). Besides, given the relationship between septic cardiomyocytes dysfunction and several apoptotic indexes, western blot analysis showed that the protective effect of silencing Pellino1 on septic cardiomyocytes dysfunction was mediated by reduced cleaved caspase-3 and Bax expression but not increased Bcl2 expression (Fig. 1g–i). In addition, LPS-induced inflammatory cytokine release was partially abolished by silencing Pellino1. Collectively, these data suggest that inhibition of Pellino1 attenuates LPS-induced cardiomyocytes dysfunction and promotes survival (Fig. 1j).

### Silencing Pellino1 alleviates the effects of LPS on cardiac fuel and energy metabolism

**Fig. 2:**
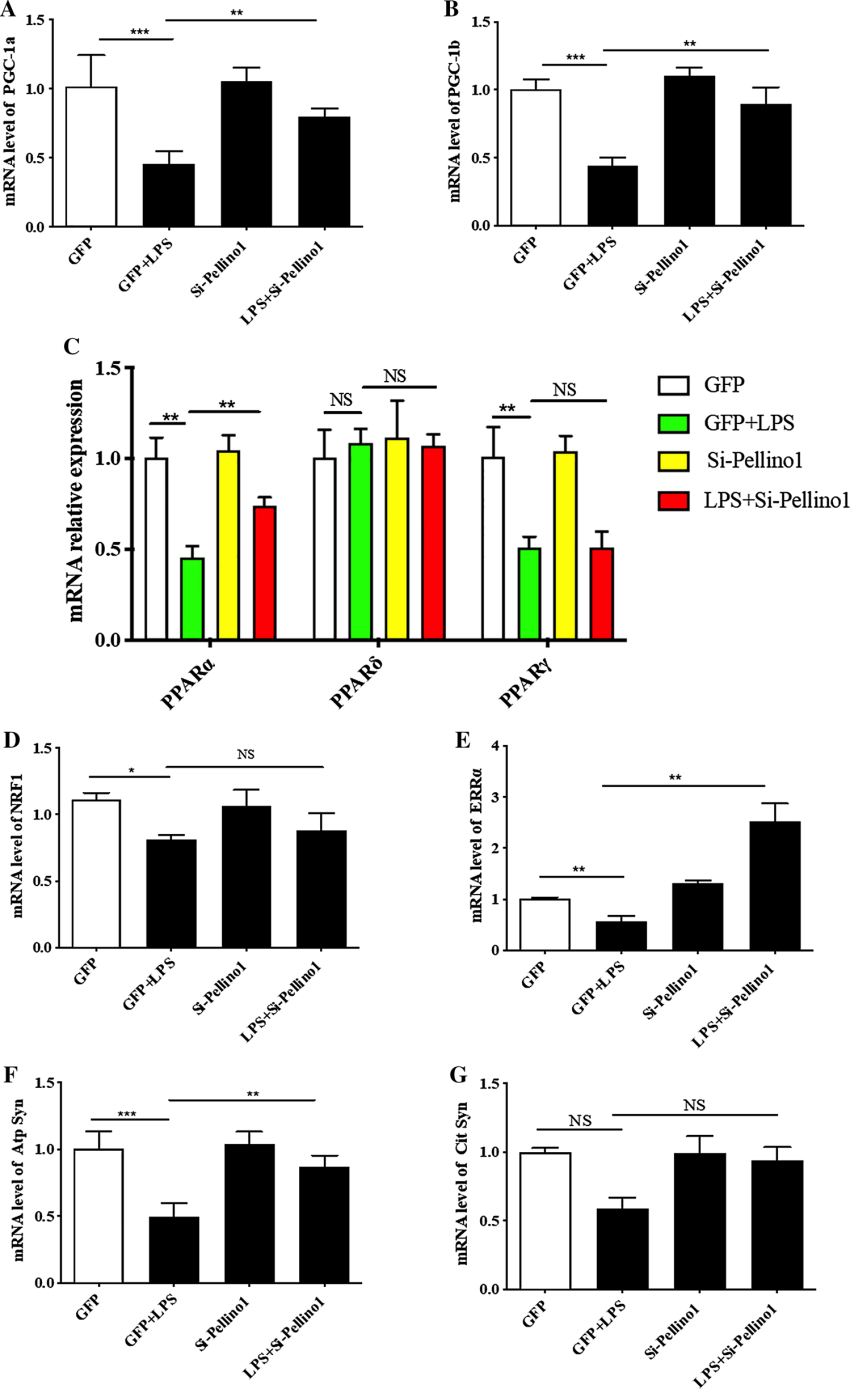
Silencing Pellino1 alleviates the effects of LPS on cardiac mitochondrial function. **a**–**e** Representative qRT-PCR of PGC-1α, PGC-1β, PPARα, PPARγ, PPARδ, NRF1, and ERRα mRNA expression following LPS treatment after Pellino1 silencing. **f**, **g** qRT-PCR validation of downstream metabolic genes of PGC-1 coactivators, including ATP Synthase β (Atp5b) and Citrate Synthase (Cit syn), involved in mitochondrial function. All mRNA expression was normalized to that of GAPDH. *n* = 6, results are expressed as the mean ± SD, *P < 0.05, **P < 0.01, ***P < 0.001, compared with the GFP group

**Fig. 3:**
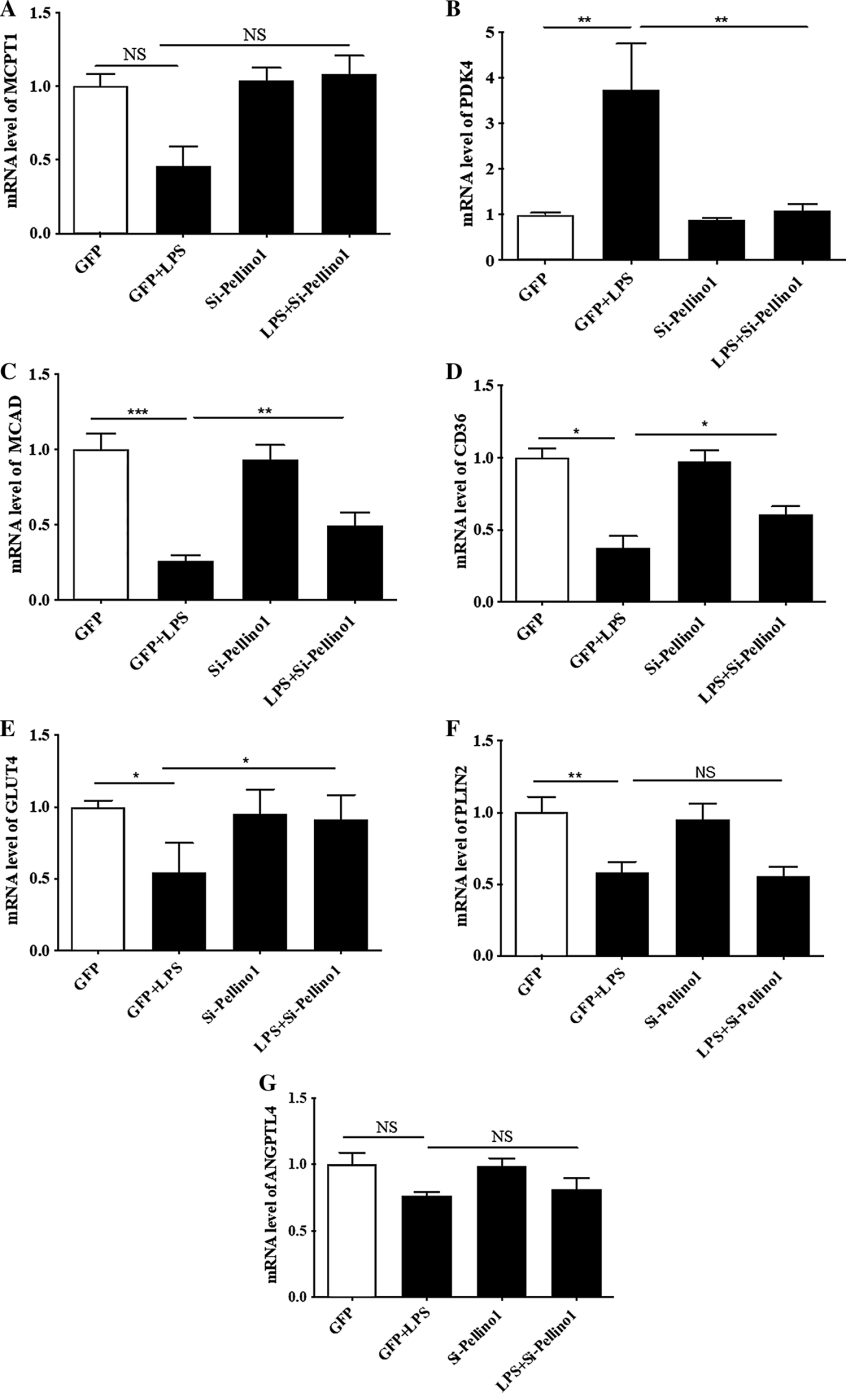
Silencing Pellino1 alleviates the effects of LPS on cardiac fuel metabolism. **a**–**d** qRT-PCR validation of downstream metabolic genes of PGC-1 coactivators, including MCPT-1 (Cpt1b), PDK4, MCAD (Acadm), and CD36 involved in fatty acid β-oxidation (FAO). **e** Representative qRT-PCR of the mRNA expression of GLUT4 (Slc2a4), the PGC-1 target gene encoding the glucose transporter, followed by Pellino1-silencing adenovirus treatment. **f**, **g** Representative qRT-PCR of the mRNA expression of PLIN2 and angiopoietin-like 4 (ANGPTL4) involved in the regulation of FAO and myocardial lipid metabolism, respectively, following LPS treatment after Pellino1 silencing. All mRNA expression was normalized to GAPDH. *n* = 6, results are expressed as the mean ± SD, *P < 0.05, **P < 0.01, ***P < 0.001, compared with the GFP group

Given the regulatory role of PGC-1 coactivators in maintaining a high-capacity mitochondrial system, we next assessed the effect of silencing Pellino1 on the myocardial expression of LPS-induced cardiac PGC-1α and β genes. RT-PCR showed that LPS-mediated rapid downregulation of the PGC-1α and β mRNA levels was preserved in the Pellino1-silenced cells (Fig. 2a, b). The LPS-induced reduction of the PGC-1 coactivators that mediate metabolic transcription factors [PPARα, ERRα, NRF1 (Nrf1)] was partially reversed by silencing Pellino1 (Fig. 2c–e). However, silencing Pellino1 had no effect on the expression of PPARγ and PPARδ (Fig. 2c). Interestingly, the expression of downstream gene targets of PGC-1 coactivators involved in mitochondrial function [ATP Synthase β (Atp5b), Citrate Synthase (Cit syn)] and fatty acid β-oxidation (FAO) [MCPT-1 (Cpt1b), PDK4, MCAD (Acadm), CD36] was enhanced at the mRNA level following LPS treatment after silencing Pellino1 (Figs. 2f–g, 3a–d). Similar to the effects on the PGC-1 coactivator and their downstream gene targets, mRNA expression of GLUT4 (Slc2a4), the PGC-1 target gene encoding the glucose transporter, was also increased by silencing Pellino1 through adenovirus treatment (Fig. 3e). In addition, other genes involved in the regulation of FAO, such as PLIN2 and the myocardial lipid metabolic gene angiopoietin-like 4 (ANGPTL4), were not differentially expressed in the inflamed NRCMs and the si-Pellino1 group (Fig. 3f, g). Collectively, these data support the view that LPS-mediated inflammation leads to rapid deactivation of the PGC-1 metabolic gene regulatory circuit, and Pellino1 may be a potential target for cardiac fuel and energy metabolism.

### LC–MS-mediated metabolomics analysis of LPS-treated or Pellino1-silenced NRCMs

**Fig. 4:**
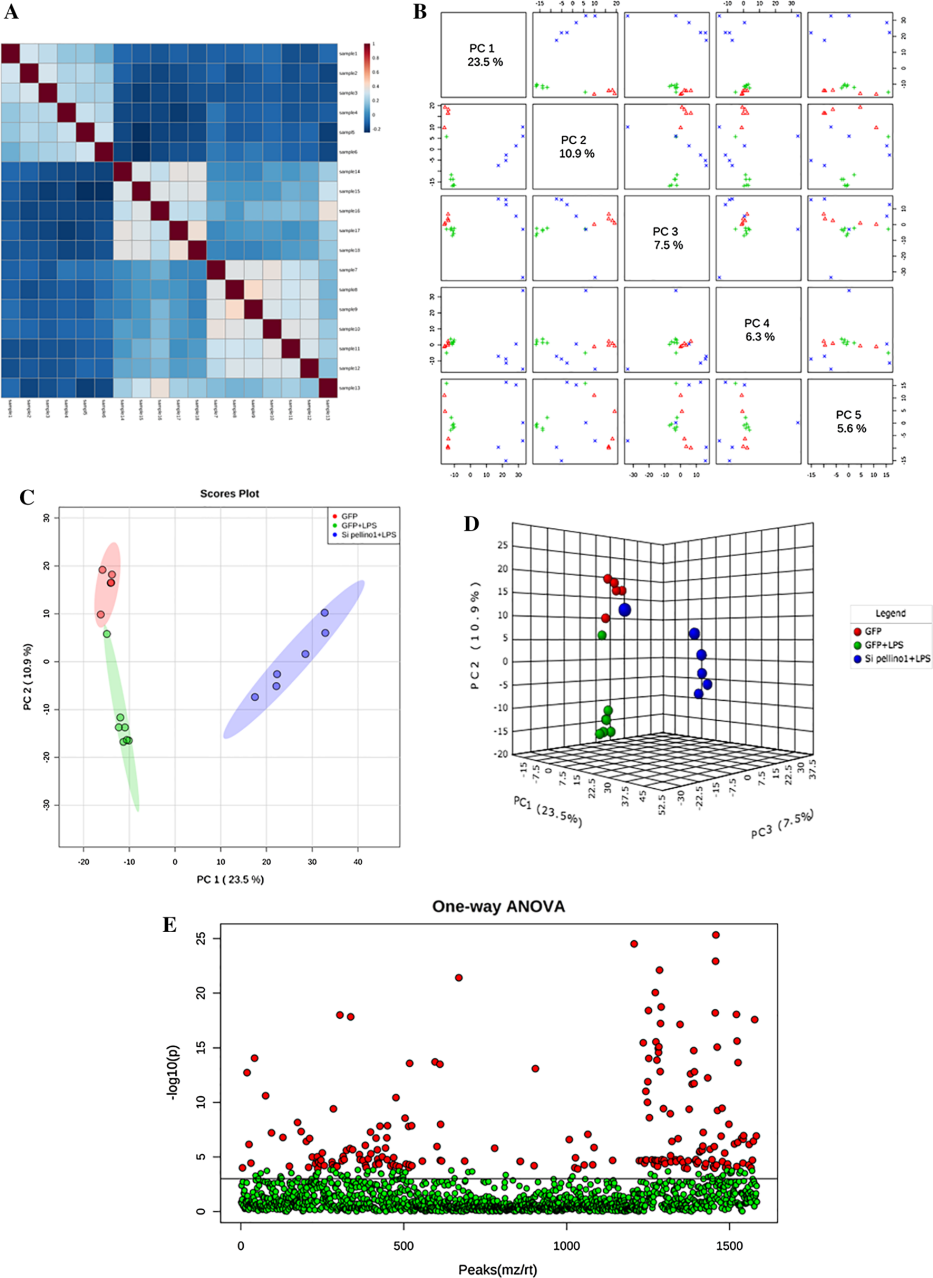
LC–MS-mediated metabolomics analysis of LPS-treated or Pellino1-silenced NRCMs. **a** Correlation analysis identified good sequencing stability and negative associations among samples. **b** Different perspectives of two-dimensional principal components (PC) showed the variable reduction outputs analyzed by orthogonal partial least-squares projections to latent structures-discriminant analyses (OPLS-DA) on metabolites from si-Pellino1 and LPS-treated, LPS-treated, and untreated control NRCMs. **c**, **d** OPLS-DA score plots of three groups (GFP, GFP + LPS, si-Pellino1 + LPS) according to the differential metabolic profile showed in 2D (**c**) and 3D (**d**); each point represents a tested sample. **e** One-way ANOVA (*analysis* of variation) of significant levels of all 169 metabolites, *P* < 0.05

To identify metabolite alterations that are induced by LPS or silencing Pellino1 adenovirus, we used LC–MS to analyze and evaluate all samples. First, the total ion flow diagram of the analyzed QC samples was compared with the spectral overlap by a comparison of the QC sample spectrogram. The results showed that the response strength and retention time of each chromatographic peak overlapped, indicating that the variation caused by instrument error was small in the whole experiment (Fig. S1A). Then, metabolic profiling (Chenomx, Edmonton, AB, Canada) was used to identify 169 differentially abundant metabolites between the LPS-treated cells and the untreated control cells (Table 1). After normalization of the data, univariate and multivariate statistical analyses were used to comprehensively evaluate the effects of silencing Pellino1 on the LPS-treated NRCMs (Figs. S1B–E, S2A–D). Our correlation analysis identified good sequencing stability and negative associations among the samples and metabolites (Fig. 4a).

**Table 1:** One-way ANOVA (analysis of variation) test of significant levels of all 169 metabolites

Name	*f* value	*P* value	− log10(*P*)	FDR	Tukey’s HSD	HMDB	PubChem	KEGG
d-glucose	8.6856	0.0031225	2.5055	0.017393	gfp + lps-293 + lps; gfp + lps-gfp	HMDB0000122	5793	C00031
l-leucine	6.1263	0.011353	1.9449	0.045644	gfp + lps-293 + lps; gfp + lps-gfp	HMDB0000687	6106	C00123
l-palmitoylcarnitine	22.18	3.31E−05	4.4805	0.00038715	gfp + lps-293 + lps; gfp + lps-gfp	HMDB0013336	53481691	
l-tyrosine	19.542	6.65E−05	4.1773	0.00067306	gfp + lps-293 + lps; gfp + lps-gfp	HMDB0000158	6057	C00082
N10-formyl-THF	8.0235	0.0042711	2.3695	0.02162	gfp + lps-293 + lps; gfp + lps-gfp	HMDB0000972	122347	C00234
(2E)-decenoyl-CoA	10.772	0.0012575	2.9005	0.0085285	gfp + lps-293 + lps; gfp + lps-gfp	HMDB0003948	5280768	C05275
(2E)-hexadecenoyl-CoA	14.908	0.00027227	3.565	0.0022887	gfp + lps-293 + lps; gfp + lps-gfp	HMDB0003945	46173176	C05272
(S)-3-hydroxybutanoyl-CoA	8.0514	0.004214	2.3753	0.021528	gfp + lps-293 + lps; gfp + lps-gfp	HMDB0001166	440045	C03561
(*S*)-methylmalonic acid semialdehyde	578.91	6.33E−15	14.199	8.36E−13	gfp + lps-293 + lps; gfp + lps-gfp	HMDB0002217	5462303	C06002
1-Methylhistidine	13.709	0.00041119	3.386	0.0032713	gfp + lps-293 + lps; gfp + lps-gfp	HMDB0000001	92105	C01152
1-Pyrroline-2-carboxylic acid	7.4121	0.0057732	2.2386	0.02687	gfp + lps-293 + lps; gfp + lps-gfp	HMDB0006875	440046	C03564
2,3-Diphosphoglyceric acid	14.163	0.00035084	3.4549	0.0028624	gfp + lps-293 + lps; gfp + lps-gfp	HMDB0001294	186004	C01159
2-Acyl-sn-glycero-3-phosphocholine	11.256	0.0010338	2.9856	0.0072786	gfp + lps-293 + lps; gfp + lps-gfp	METPA0476	NA	C04233
3-Carboxy-1-hydroxypropylthiamine diphosphate	12.331	0.00068068	3.1671	0.0050622	gfp + lps-293 + lps; gfp + lps-gfp	HMDB0006744	440649	C05381
3-Dehydro-l-gulonate	6.1974	0.010919	1.9618	0.044347	gfp + lps-293 + lps; gfp + lps-gfp	HMDB0006334	439273	C00618
3-Hydroxy-3-methylglutaryl-CoA	7.6277	0.005184	2.2853	0.024709	gfp + lps-293 + lps; gfp + lps-gfp	HMDB0001375	439218	C00356
3-Hydroxyanthranilic acid	20.579	5.01E−05	4.2998	0.00054116	gfp + lps-293 + lps; gfp + lps-gfp	HMDB0001476	86	C00632
3-Methyl-2-oxovaleric acid	18.168	9.83E−05	4.0074	0.0009275	gfp + lps-293 + lps; gfp + lps-gfp	HMDB0000491	47	C03465
3-Oxooctanoyl-CoA	1227.3	2.38E−17	16.624	3.30E−14	gfp + lps-293 + lps; gfp + lps-gfp	HMDB0003941	440608	C05267
4-(2-Aminophenyl)-2,4-dioxobutanoic acid	27.45	9.71E−06	5.0129	0.00015386	gfp + lps-293 + lps; gfp + lps-gfp	HMDB0000978	472	C01252
4-Aminobutyraldehyde	7.2007	0.0064257	2.1921	0.029221	gfp + lps-293 + lps; gfp + lps-gfp	HMDB0001080	118	C00555
4-Fumarylacetoacetic acid	14.574	0.00030472	3.5161	0.0025157	gfp + lps-293 + lps; gfp + lps-gfp	HMDB0001268	5280398	C01061
4-Methyl-2-oxopentanoate	9.0251	0.0026722	2.5731	0.015516	gfp + lps-293 + lps; gfp + lps-gfp	HMDB0000695	70	C00233
5,10-Methenyltetrahydrofolic acid	11.204	0.0010556	2.9765	0.0073946	gfp + lps-293 + lps; gfp + lps-gfp	HMDB0001354	644350	C00445
5,10-Methylene-THF	8.323	0.0037007	2.4317	0.019554	gfp + lps-293 + lps; gfp + lps-gfp	HMDB0001533	439175	C00143
5-Aminolevulinic acid	29.971	5.76E−06	5.2398	9.39E−05	gfp + lps-293 + lps; gfp + lps-gfp	HMDB0001149	137	C00430
5-Hydroxy-l-tryptophan	7.1572	0.0065702	2.1824	0.029781	gfp + lps-293 + lps; gfp + lps-gfp	HMDB0000472	144	C01017
5′-Methylthioadenosine	16.604	0.00015751	3.8027	0.0013871	gfp + lps-293 + lps; gfp + lps-gfp	HMDB0001173	439176	C00170
Acetaldehyde	10.331	0.0015109	2.8208	0.0099788	gfp + lps-293 + lps; gfp + lps-gfp	HMDB0000990	177	C00084
Acetic acid	56.369	1.06E−07	6.9767	2.30E−06	gfp + lps-293 + lps; gfp + lps-gfp	HMDB0000042	176	C00033
Acetoacetyl-CoA	6.6609	0.0085071	2.0702	0.036622	gfp + lps-293 + lps; gfp + lps-gfp	HMDB0001484	439214	C00332
Acetylcholine	28.233	8.22E−06	5.0851	0.00013182	gfp + lps-293 + lps; gfp + lps-gfp	HMDB0000895	187	C01996
Acetyl-CoA	11.606	9.00E−04	3.0458	0.0065356	gfp + lps-293 + lps; gfp + lps-gfp	HMDB0001206	444493	C00024
Acrylyl-CoA	12.757	0.00058031	3.2363	0.0044716	gfp + lps-293 + lps; gfp + lps-gfp	HMDB0002307	439340	C00894
Aldehyde	19.468	6.79E−05	4.1684	0.00068456	gfp + lps-293 + lps; gfp + lps-gfp	HMDB0000990	177	C00084
Alpha-d-glucose	7.497	0.0055327	2.2571	0.02619	gfp + lps-293 + lps; gfp + lps-gfp	HMDB0003345	79025	C00267
Alpha-ketoisovaleric acid	25.967	1.34E−05	4.8716	0.00020827	gfp + lps-293 + lps; gfp + lps-gfp	HMDB0000019	49	C00141
Alpha-lactose	11.104	0.0010986	2.9592	0.0076573	gfp + lps-293 + lps; gfp + lps-gfp	HMDB0000186	84571	C00243
Beta-alanine	6.4949	0.0092934	2.0318	0.039195	gfp + lps-293 + lps; gfp + lps-gfp	HMDB0000056	239	C00099
Beta-d-fructose 6-phosphate	10.953	0.0011683	2.9325	0.0080071	gfp + lps-293 + lps; gfp + lps-gfp	HMDB0003971	440641	C05345
Beta-d-glucose	19.674	6.41E−05	4.1932	0.00065605	gfp + lps-293 + lps; gfp + lps-gfp	HMDB0000516	64689	C00221
Beta-d-glucose 6-phosphate	11.014	0.0011396	2.9432	0.0078641	gfp + lps-293 + lps; gfp + lps-gfp	HMDB0003498	439427	C01172
Betaine	7.8836	0.0045713	2.34	0.022718	gfp + lps-293 + lps; gfp + lps-gfp	HMDB0000043	247	C00719
Carbamoylphosphate	9.0239	0.0026737	2.5729	0.015516	gfp + lps-293 + lps; gfp + lps-gfp	HMDB0001096	278	C00169
Carnosine	9.8813	0.0018295	2.7377	0.011775	gfp + lps-293 + lps; gfp + lps-gfp	HMDB0000033	439224	C00386
CDP-diacylglycerol	16.605	0.00015747	3.8028	0.0013871	gfp + lps-293 + lps; gfp + lps-gfp	METPA0023	NA	C00269
Choline	9.5656	0.0020992	2.6779	0.012687	gfp + lps-293 + lps; gfp + lps-gfp	HMDB0000097	305	C00114
*cis*-aconitic acid	6.2363	0.010689	1.9711	0.043668	gfp + lps-293 + lps; gfp + lps-gfp	HMDB0000072	643757	C00417
Citicoline	441.5	4.69E−14	13.329	4.34E−12	gfp + lps-293 + lps; gfp + lps-gfp	HMDB0001413	13804	C00307
Citric acid	9.8495	0.0018548	2.7317	0.011801	gfp + lps-293 + lps; gfp + lps-gfp	HMDB0000094	311	C00158
Citrulline	9.7583	0.0019296	2.7145	0.01211	gfp + lps-293 + lps; gfp + lps-gfp	HMDB0000904	9750	C00327
Coenzyme A	6.5841	0.0088612	2.0525	0.037759	gfp + lps-293 + lps; gfp + lps-gfp	HMDB0001423	6816	C00010
Creatine	9.5997	0.002068	2.6844	0.012558	gfp + lps-293 + lps; gfp + lps-gfp	HMDB0000064	586	C00300
Crotonoyl-CoA	34.922	2.27E−06	5.644	3.96E−05	gfp + lps-293 + lps; gfp + lps-gfp	HMDB0002009	5280381	C00877
Cysteinylglycine	6.7465	0.0081309	2.0899	0.035353	gfp + lps-293 + lps; gfp + lps-gfp	HMDB0000078	439498	C01419
d-aspartic acid	5.9436	0.012563	1.9009	0.049363	gfp + lps-293 + lps; gfp + lps-gfp	HMDB0006483	83887	C00402
Dephospho-CoA	119.84	5.97E−10	9.2242	2.30E−08	gfp + lps-293 + lps; gfp + lps-gfp	HMDB0001373	439335	C00882
d-galactose	14.738	0.00028826	3.5402	0.0023941	gfp + lps-293 + lps; gfp + lps-gfp	HMDB0000143	439357	C00984
d-glucuronic acid	11.051	0.0011227	2.9497	0.0077859	gfp + lps-293 + lps; gfp + lps-gfp	HMDB0000127	444791	C00191
d-glyceraldehyde 3-phosphate	8.0458	0.0042253	2.3741	0.021546	gfp + lps-293 + lps; gfp + lps-gfp	HMDB0001112	729	C00661
d-mannose	8.927	0.0027943	2.5537	0.016047	gfp + lps-293 + lps; gfp + lps-gfp	HMDB0000169	18950	C00159
d-pantothenoyl-l-cysteine	6.0625	0.01176	1.9296	0.047076	gfp + lps-293 + lps; gfp + lps-gfp	HMDB0006834	440217	C04079
d-proline	13.806	0.00039732	3.4009	0.0031855	gfp + lps-293 + lps; gfp + lps-gfp	HMDB0003411	8988	C00763
d-ribulose 5-phosphate	201.8	1.44E−11	10.843	7.24E−10	gfp + lps-293 + lps; gfp + lps-gfp	HMDB0000618	439184	C00199
d-tagatose 6-phosphate	17.257	0.0001289	3.8897	0.0011686	gfp + lps-293 + lps; gfp + lps-gfp	HMDB0006873	439396	C01097
d-xylitol	6.1641	0.01112	1.9539	0.0449	gfp + lps-293 + lps; gfp + lps-gfp	HMDB0002917	6912	C00379
Enzyme N6-(dihydrolipoyl)lysine	16.376	0.00016915	3.7717	0.0014755	gfp + lps-293 + lps; gfp + lps-gfp	METPA1231	NA	C15973
Epinephrine	8.5607	0.0033093	2.4803	0.01786	gfp + lps-293 + lps; gfp + lps-gfp	HMDB0000068	5816	C00788
Formic acid	7.8027	0.0047555	2.3228	0.023307	gfp + lps-293 + lps; gfp + lps-gfp	HMDB0000142	284	C00058
Formiminoglutamic acid	59.932	7.02E−08	7.1535	1.59E−06	gfp + lps-293 + lps; gfp + lps-gfp	HMDB0000854	439233	C00439
Fumaric acid	24.319	1.96E−05	4.7071	0.0002821	gfp + lps-293 + lps; gfp + lps-gfp	HMDB0000134	444972	C00122
Galactinol	9.5262	0.0021359	2.6704	0.012825	gfp + lps-293 + lps; gfp + lps-gfp	HMDB0005826	439451	C01235
Gamma-aminobutyric acid	7.5207	0.0054675	2.2622	0.025941	gfp + lps-293 + lps; gfp + lps-gfp	HMDB0000112	119	C00334
Gamma-glutamylcysteine	11.334	0.0010021	2.9991	0.0071097	gfp + lps-293 + lps; gfp + lps-gfp	HMDB0001049	123938	C00669
Glucose 1-phosphate	17.842	0.00010817	3.9659	0.0010035	gfp + lps-293 + lps; gfp + lps-gfp	HMDB0001586	439165	C00103
Glucose 6-phosphate	8.2948	0.0037505	2.4259	0.019746	gfp + lps-293 + lps; gfp + lps-gfp	HMDB0001401	5958	C00668
Glutaryl-CoA	20.788	4.74E−05	4.324	0.00051588	gfp + lps-293 + lps; gfp + lps-gfp	HMDB0001339	439252	C00527
Glutathione	8.6649	0.0031526	2.5013	0.017468	gfp + lps-293 + lps; gfp + lps-gfp	HMDB0000125	124886	C00051
Glyceric acid	8.5039	0.0033983	2.4687	0.018161	gfp + lps-293 + lps; gfp + lps-gfp	HMDB0000139	439194	C00258
Sarcosine	7.1053	0.0067472	2.1709	0.030533	gfp + lps-gfp	HMDB0000271	1088	C00213
Serotonin	6.2557	0.010577	1.9757	0.043466	gfp + lps-gfp	HMDB0000259	5202	C00780
Sorbitol	6.7612	0.0080686	2.0932	0.035303	gfp + lps-gfp	HMDB0000247	5780	C00794
Spermidine	6.9465	0.007324	2.1353	0.032521	gfp + lps-gfp	HMDB0001257	1102	C00315
Spermine	5.9836	0.012286	1.9106	0.048618	gfp + lps-gfp	HMDB0001256	1103	C00750
Stachyose	11.466	0.00095083	3.0219	0.0068635	gfp + lps-gfp	HMDB0003553	439531	C01613
Succinic acid	7.0847	0.0068193	2.1663	0.030659	gfp + lps-gfp	HMDB0000254	1110	C00042
Succinyl-CoA	6.4644	0.0094469	2.0247	0.039766	gfp + lps-gfp	HMDB0001022	439161	C00091
Sucrose	6.9038	0.0074884	2.1256	0.033025	gfp + lps-gfp	HMDB0000258	5988	C00089
Tetrahydrofolic acid	8.0685	0.0041793	2.3789	0.02139	gfp + lps-gfp	HMDB0001846	91443	C00101
*trans*-2-hexenoyl-CoA	6.1165	0.011415	1.9425	0.045826	gfp + lps-gfp	HMDB0003944	5280765	C05271
Tryptamine	6.2667	0.010513	1.9783	0.04327	gfp + lps-gfp	HMDB0000303	1150	C00398
Tyramine	6.6133	0.0087246	2.0593	0.037349	gfp + lps-gfp	HMDB0000306	5610	C00483
Uracil	7.0882	0.0068069	2.1671	0.030653	gfp + lps-gfp	HMDB0000300	1174	C00106
Ureidosuccinic acid	7.3834	0.0058572	2.2323	0.02708	gfp + lps-gfp	HMDB0000828	93072	C00438
Uridine diphosphate glucuronic acid	6.0717	0.0117	1.9318	0.046903	gfp + lps-gfp	HMDB0000935	17473	C00167
Xylulose 5-phosphate	6.5547	0.0090011	2.0457	0.038296	gfp + lps-gfp	HMDB0000868	439190	C00231
3-Oxododecanoyl-CoA	6.6288	0.0086531	2.0628	0.037157	gfp + lps-gfp	HMDB0003937	440604	C05263
Butanoyl-CoA	6.014	0.012081	1.9179	0.048149	gfp + lps-gfp	HMDB0001088	265	C00136
*cis*,*cis*-3,6-Dodecadienoyl-CoA	7.3222	0.0060409	2.2189	0.027744	gfp + lps-gfp	HMDB0003952	5280771	C05280
(S)-3-hydroxydodecanoyl-CoA	10.951	0.001169	2.9322	0.0080071	gfp + lps-gfp	HMDB0003936	440603	C05262
(S)-3-hydroxytetradecanoyl-CoA	6.3041	0.010301	1.9871	0.042524	gfp + lps-gfp	HMDB0003934	440602	C05260
3-Oxohexanoyl-CoA	7.4828	0.005572	2.254	0.026226	gfp + lps-gfp	HMDB0003943	440610	C05269
3-Oxotetradecanoyl-CoA	6.4356	0.0095944	2.018	0.040204	gfp + lps-gfp	HMDB0003935	11966197	C05261
3-Oxohexadecanoyl-CoA	6.0107	0.012103	1.9171	0.048168	gfp + lps-gfp	HMDB0006402	440601	C05259
l-palmitoylcarnitine	9.9708	0.0017604	2.7544	0.011463	gfp + lps-gfp	HMDB0000222	11953816	C02990
Glyceric acid 1,3-biphosphate	26.235	1.27E−05	4.8977	0.00019837	gfp-293 + lps; gfp + lps-293 + lps; gfp + lps-gfp	HMDB0001270	683	C00236
Glycerol 3-phosphate	30.839	4.85E−06	5.3144	8.01E−05	gfp-293 + lps; gfp + lps-293 + lps; gfp + lps-gfp	HMDB0000126	439162	C00093
Glycerophosphocholine	443.56	4.53E−14	13.344	4.33E−12	gfp-293 + lps; gfp + lps-293 + lps; gfp + lps-gfp	HMDB0000086	71920	C00670
Glycerylphosphorylethanolamine	22.141	3.34E−05	4.4763	0.00038928	gfp-293 + lps; gfp + lps-293 + lps; gfp + lps-gfp	HMDB0000114	22833510	C01233
Glycolic acid	483.97	2.38E−14	13.623	2.69E−12	gfp-293 + lps; gfp + lps-293 + lps; gfp + lps-gfp	HMDB0000115	757	C00160
Hexanoyl-CoA	557.17	8.40E−15	14.075	1.06E−12	gfp-293 + lps; gfp + lps-293 + lps; gfp + lps-gfp	HMDB0002845	440611	C05270
Hydroxyproline	22.869	2.78E−05	4.5553	0.00034027	gfp-293 + lps; gfp + lps-293 + lps; gfp + lps-gfp	HMDB0000725	5810	C01157
Hydroxypyruvic acid	859.78	3.36E−16	15.473	1.33E−13	gfp-293 + lps; gfp + lps-293 + lps; gfp + lps-gfp	HMDB0001352	964	C00168
Imidazoleacetic acid	69.118	2.69E−08	7.5695	7.05E−07	gfp-293 + lps; gfp + lps-293 + lps; gfp + lps-gfp	HMDB0002024	96215	C02835
Indoleacetaldehyde	167.59	5.48E−11	10.262	2.46E−09	gfp-293 + lps; gfp + lps-293 + lps; gfp + lps-gfp	HMDB0001190	800	C00637
Indoleacetic acid	44.274	5.09E−07	6.2929	9.95E−06	gfp-293 + lps; gfp + lps-293 + lps; gfp + lps-gfp	HMDB0000197	802	C00954
Isocitric acid	75.679	1.45E−08	7.8372	3.96E−07	gfp-293 + lps; gfp + lps-293 + lps; gfp + lps-gfp	HMDB0000193	1198	C00311
l-alanine	120.66	5.68E−10	9.2453	2.22E−08	gfp-293 + lps; gfp + lps-293 + lps; gfp + lps-gfp	HMDB0000161	5950	C00041
l-arabitol	57.583	9.16E−08	7.0381	2.02E−06	gfp-293 + lps; gfp + lps-293 + lps; gfp + lps-gfp	HMDB0001851	439255	C00532
l-arginine	439.18	4.87E−14	13.312	4.36E−12	gfp-293 + lps; gfp + lps-293 + lps; gfp + lps-gfp	HMDB0000517	6322	C00062
l-asparagine	134.61	2.62E−10	9.5816	1.05E−08	gfp-293 + lps; gfp + lps-293 + lps; gfp + lps-gfp	HMDB0000168	6267	C00152
l-aspartic acid	17.315	0.00012666	3.8974	0.001152	gfp-293 + lps; gfp + lps-293 + lps; gfp + lps-gfp	HMDB0000191	5960	C00049
Lauroyl-CoA	623.05	3.67E−15	14.435	6.37E−13	gfp-293 + lps; gfp + lps-293 + lps; gfp + lps-gfp	HMDB0003571	165436	C01832
l-cysteine	248.05	3.21E−12	11.493	1.82E−10	gfp-293 + lps; gfp + lps-293 + lps; gfp + lps-gfp	HMDB0000574	5862	C00097
l-dopa	119.6	6.05E−10	9.2181	2.30E−08	gfp-293 + lps; gfp + lps-293 + lps; gfp + lps-gfp	HMDB0000181	6047	C00355
l-dopachrome	104.33	1.58E−09	8.8014	5.41E−08	gfp-293 + lps; gfp + lps-293 + lps; gfp + lps-gfp	HMDB0001430	439549	C01693
l-glutamic acid	95.038	3.03E−09	8.5187	1.01E−07	gfp-293 + lps; gfp + lps-293 + lps; gfp + lps-gfp	HMDB0000148	33032	C00025
l-glutamic-gamma-semialdehyde	172.9	4.38E−11	10.359	2.06E−09	gfp-293 + lps; gfp + lps-293 + lps; gfp + lps-gfp	HMDB0002104	193305	C01165
l-histidine	45.975	4.00E−07	6.3982	8.02E−06	gfp-293 + lps; gfp + lps-293 + lps; gfp + lps-gfp	HMDB0000177	6274	C00135
l-homocysteine	34.755	2.34E−06	5.6311	4.05E−05	gfp-293 + lps; gfp + lps-293 + lps; gfp + lps-gfp	HMDB0000742	778	C00155
l-isoleucine	28.382	7.97E−06	5.0986	0.00012852	gfp-293 + lps; gfp + lps-293 + lps; gfp + lps-gfp	HMDB0000172	6306	C00407
l-lactic acid	36.494	1.73E−06	5.7625	3.07E−05	gfp-293 + lps; gfp + lps-293 + lps; gfp + lps-gfp	HMDB0000190	107689	C00186
l-malic acid	469.31	2.99E−14	13.525	3.19E−12	gfp-293 + lps; gfp + lps-293 + lps; gfp + lps-gfp	HMDB0000156	222656	C00149
l-proline	208.07	1.15E−11	10.939	5.91E−10	gfp-293 + lps; gfp + lps-293 + lps; gfp + lps-gfp	HMDB0000162	145742	C00148
l-threonine	608.13	4.40E−15	14.357	6.70E−13	gfp-293 + lps; gfp + lps-293 + lps; gfp + lps-gfp	HMDB0000167	6288	C00188
l-tryptophan	19.926	5.98E−05	4.2233	0.00062363	gfp-293 + lps; gfp + lps-293 + lps; gfp + lps-gfp	HMDB0000929	6305	C00078
Glucosamine 6-phosphate	36.93	1.60E−06	5.7946	2.89E−05	gfp-293 + lps; gfp + lps-293 + lps; gfp + lps-gfp	HMDB0001254	439217	C00352
l-valine	22.97	2.72E−05	4.566	0.00033788	gfp-293 + lps; gfp + lps-293 + lps; gfp + lps-gfp	HMDB0000883	6287	C00183
Maleylacetoacetic acid	18.897	7.97E−05	4.0987	0.00077816	gfp-293 + lps; gfp + lps-293 + lps; gfp + lps-gfp	HMDB0002052	5280393	C01036
Malonyl-CoA	52.738	1.64E−07	6.7861	3.49E−06	gfp-293 + lps; gfp + lps-293 + lps; gfp + lps-gfp	HMDB0001175	10663	C00083
Melatonin	76.15	1.39E−08	7.8555	3.83E−07	gfp-293 + lps; gfp + lps-293 + lps; gfp + lps-gfp	HMDB0001389	896	C01598
Methylmalonic acid	53.513	1.49E−07	6.8277	3.20E−06	gfp-293 + lps; gfp + lps-293 + lps; gfp + lps-gfp	HMDB0000202	487	C02170
Myoinositol	41.927	7.21E−07	6.1418	1.39E−05	gfp-293 + lps; gfp + lps-293 + lps; gfp + lps-gfp	HMDB0000211	NA	C00137
*N*-acetyl-l-alanine	21.608	3.83E−05	4.4172	0.00043152	gfp-293 + lps; gfp + lps-293 + lps; gfp + lps-gfp	HMDB0000766	88064	
*N*-acetyl-l-aspartic acid	17.908	0.0001061	3.9743	0.00099095	gfp-293 + lps; gfp + lps-293 + lps; gfp + lps-gfp	HMDB0000812	65065	C01042
*N*-acetylserotonin	25.25	1.58E−05	4.8011	0.00024093	gfp-293 + lps; gfp + lps-293 + lps; gfp + lps-gfp	HMDB0001238	903	C00978
NADP	17.813	0.00010912	3.9621	0.001009	gfp-293 + lps; gfp + lps-293 + lps; gfp + lps-gfp	HMDB0000217	5886	C00006
NADPH	614.96	4.05E−15	14.393	6.60E−13	gfp-293 + lps; gfp + lps-293 + lps; gfp + lps-gfp	HMDB0000221	22833512	C00005
Norepinephrine	82.337	8.17E−09	8.0879	2.44E−07	gfp-293 + lps; gfp + lps-293 + lps; gfp + lps-gfp	HMDB0000216	439260	C00547
Octanoyl-CoA	25.365	1.54E−05	4.8125	0.0002373	gfp-293 + lps; gfp + lps-293 + lps; gfp + lps-gfp	HMDB0001070	380	C01944
Ornithine	61.991	5.60E−08	7.2515	1.32E−06	gfp-293 + lps; gfp + lps-293 + lps; gfp + lps-gfp	HMDB0000214	6262	C00077
Oxalacetic acid	163.73	6.47E−11	10.189	2.85E−09	gfp-293 + lps; gfp + lps-293 + lps; gfp + lps-gfp	HMDB0000223	970	C00036
Oxalosuccinic acid	36.762	1.65E−06	5.7823	2.95E−05	gfp-293 + lps; gfp + lps-293 + lps; gfp + lps-gfp	HMDB0003974	972	C05379
Oxidized glutathione	72.689	1.91E−08	7.7179	5.06E−07	gfp-293 + lps; gfp + lps-293 + lps; gfp + lps-gfp	HMDB0003337	975	C00127
Oxoglutaric acid	227.31	6.06E−12	11.217	3.17E−10	gfp-293 + lps; gfp + lps-293 + lps; gfp + lps-gfp	HMDB0000208	51	C00026
Palmityl-CoA	14.029	0.00036757	3.4347	0.0029727	gfp-293 + lps; gfp + lps-293 + lps; gfp + lps-gfp	HMDB0001338	15667	C00154
Pantetheine	64.414	4.33E−08	7.3632	1.08E−06	gfp-293 + lps; gfp + lps-293 + lps; gfp + lps-gfp	HMDB0003426	479	C00831
Pantothenic acid	132.89	2.87E−10	9.5421	1.14E−08	gfp-293 + lps; gfp + lps-293 + lps; gfp + lps-gfp	HMDB0000210	988	C00864
Phosphatidylethanolamine	109.61	1.12E−09	8.9514	3.98E−08	gfp-293 + lps; gfp + lps-293 + lps; gfp + lps-gfp	METPA0497	NA	C04438
Phosphoenolpyruvic acid	117.03	7.05E−10	9.1516	2.61E−08	gfp-293 + lps; gfp + lps-293 + lps; gfp + lps-gfp	HMDB0000263	1005	C00074
Phosphorylcholine	59.365	7.48E−08	7.126	1.67E−06	gfp-293 + lps; gfp + lps-293 + lps; gfp + lps-gfp	HMDB0001565	1014	C00588
Phosphoserine	92.746	3.59E−09	8.4451	1.16E−07	gfp-293 + lps; gfp + lps-293 + lps; gfp + lps-gfp	HMDB0000272	68841	C01005
Pyroglutamic acid	604.63	4.59E−15	14.338	6.70E−13	gfp-293 + lps; gfp + lps-293 + lps; gfp + lps-gfp	HMDB0000267	7405	C01879
Pyruvic acid	33.413	2.98E−06	5.526	5.10E−05	gfp-293 + lps; gfp + lps-293 + lps; gfp + lps-gfp	HMDB0000243	1060	C00022
Raffinose	196.92	1.71E−11	10.766	8.49E−10	gfp-293 + lps; gfp + lps-293 + lps; gfp + lps-gfp	HMDB0003213	10542	C00492
*R*-*S*-Cysteinylglycine	28.156	8.36E−06	5.0781	0.0001332	gfp-293 + lps; gfp + lps-293 + lps; gfp + lps-gfp	METPA0652	NA	C05729
*S*-acetyldihydrolipoamide-*E*	37.452	1.47E−06	5.8326	2.68E−05	gfp-293 + lps; gfp + lps-293 + lps; gfp + lps-gfp	HMDB0006878	24906332	C16255
*S*-adenosylmethioninamine	115.64	7.67E−10	9.115	2.80E−08	gfp-293 + lps; gfp + lps-293 + lps; gfp + lps-gfp	HMDB0000988	439415	C01137
*S*-adenosylmethionine	66.293	3.57E−08	7.4472	9.09E−07	gfp-293 + lps; gfp + lps-293 + lps; gfp + lps-gfp	HMDB0001185	16757548	C00019

As a multivariate statistical technique applied to determine the relative differences in two or more systems that are large and complex, principal component analysis (PCA) is the most widespread multidimensional data analysis technique, which can systematically reduce the dimensionality of original data required to describe protein dynamics with the preservation of variance. Therefore, to further calculate variable importance in projection (VIP) scores of metabolites, we performed partial least-squares projections for latent structures-discriminant analysis (PLS-DA) of metabolites induced by si-Pellino1 and LPS-treated, LPS-treated, and untreated control NRCMs. Metabolites with VIP scores greater than 1.0 were considered important. To confirm the “goodness” of the model and the predictive quality, we performed orthogonal partial least-squares projections to latent structures-discriminant analyses (OPLS-DA) on data from three groups.

The variable reduction outputs were shown from different perspectives of two-dimensional principal components (PC) contributing a percentage of the variation of a sample (Fig. 4b). As shown in Fig. 4c, d, each point represents a tested sample, and the three groups were clustered separately, suggesting that the metabolic profile was intensely influenced by the treatment with LPS and si-Pellino1. Additionally, the obvious separation among samples from different treatment groups observed in the OPLS-DA score plots according to the differential metabolic profile in each group in both positive and negative ion modes indicated that si-Pellino1 treatment led to significant changes in the metabolites compared with those of the LPS-treated control group. Interestingly, as shown in Table 1, the high − log10(*P* values) in the OPLS-DA shown in the table could reflect the differences among the three groups of samples. Furthermore, one-way ANOVA of the metabolites showed significant levels of all 169 metabolites (Fig. 4e).

### Key metabolic pathways associated with si-Pellino1 treatment

**Fig. 5:**
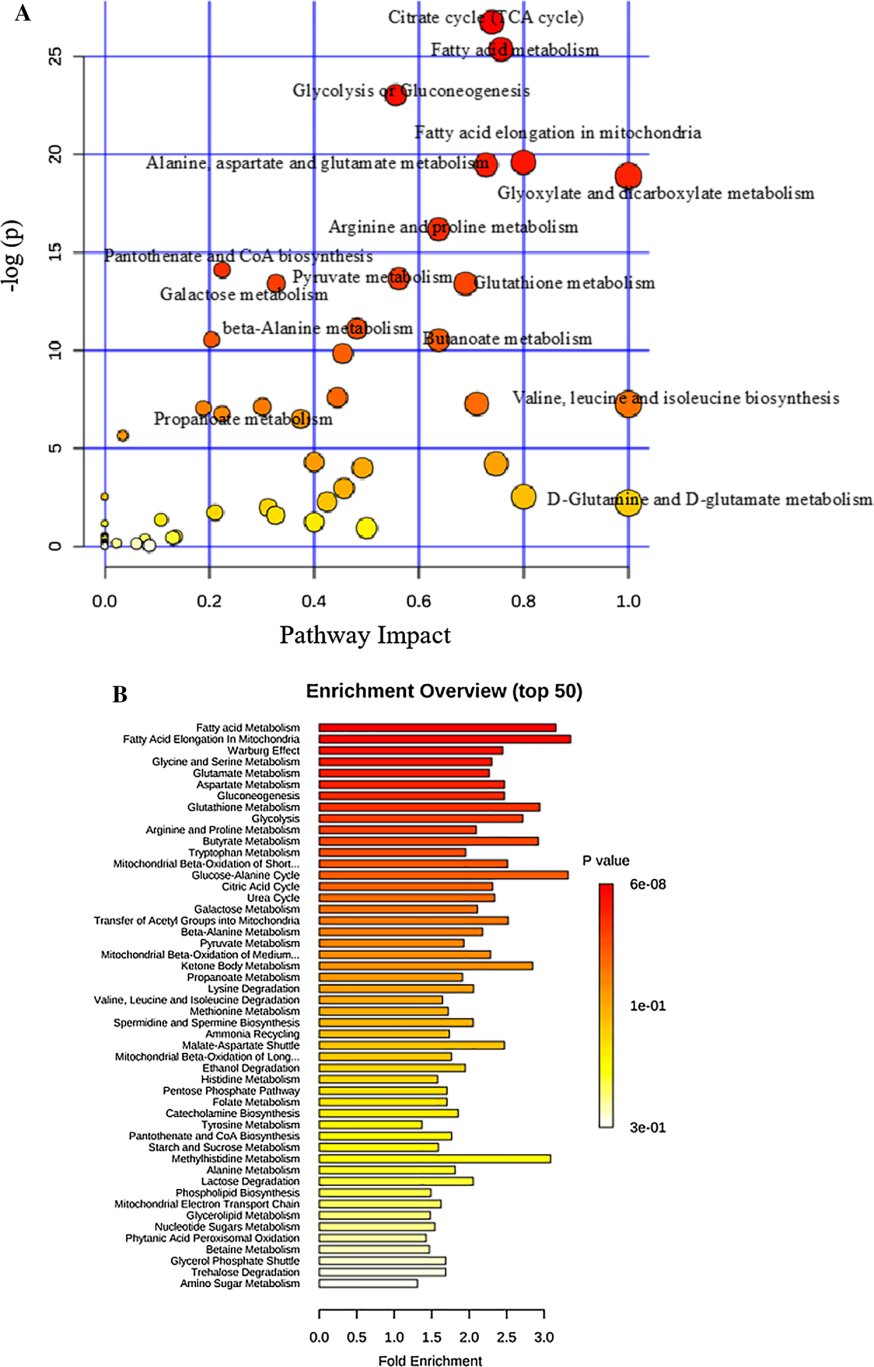
Metabolome view of the top 50 matched metabolic pathways associated with si-Pellino1 treatment. **a** Impact analysis of metabolites associated with si-Pellino1 treatment relative to the control according to *P* values (shown in Table 2) from enrichment analysis and impact values from topology analysis. The topological score (*X*-axis) and the size of each circle represent the importance of different metabolites and their impact values in the enriched pathways, while the color of each circle based on the *P* value of the enrichment analysis (*Y*-axis) represents the statistical significance of the overall metabolic changes in the pathways. **b** Enrichment analysis of metabolites associated with si-Pellino1 treatment relative to the control. The pathway from yellow to red indicates a growing impact

**Fig. 6:**
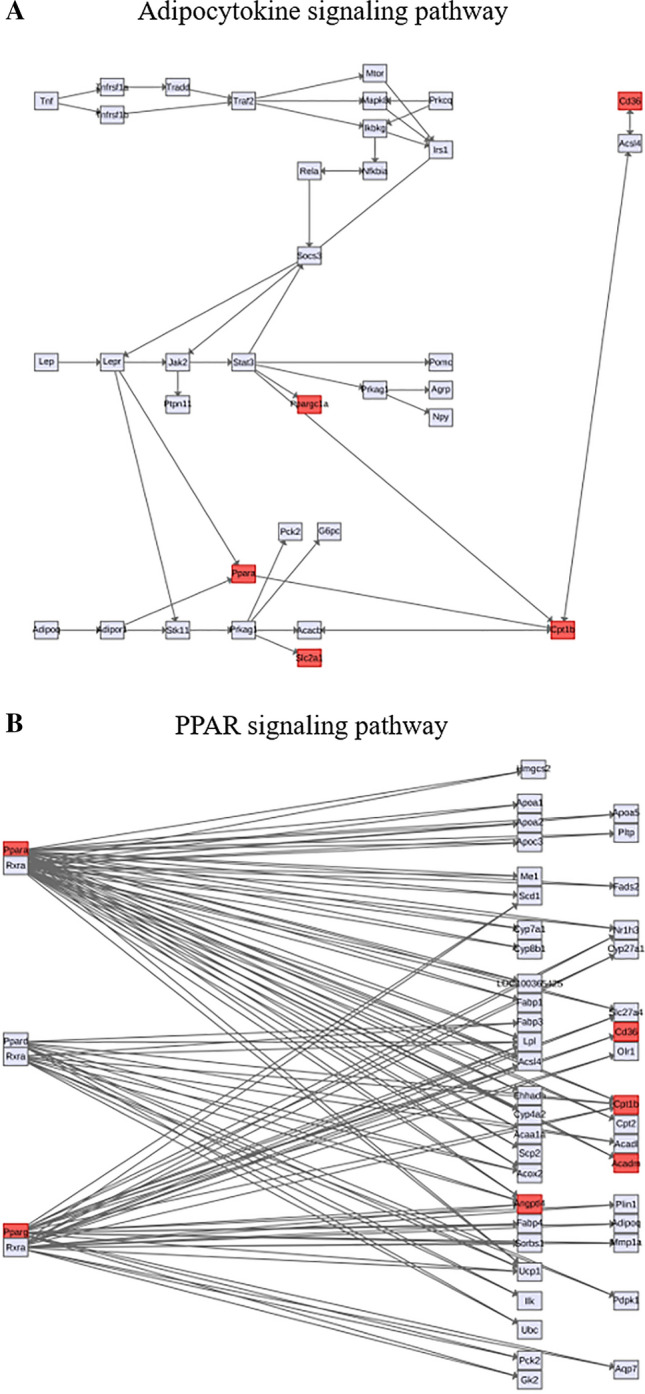
Protein interaction analysis of matched KEGG pathway associations with siPellino1 treatment. **a** Predicted protein interactions among PGC-1α, PPARα, Slc2a1, CD36, and Cpt1b involved in the adipocytokine signaling pathway. **b** Predicted protein interactions among PPARγ, PPARα, Angptl4, CD36, Cpt1b, and Acadm involved in the PPAR signaling pathway

Among the distinguishing metabolites identified by statistical correlations, to further identify which metabolic pathways were the most relevant pathways affected by si-Pellino1 treatment, we generated a metabolome view of the top 50 matched pathways according to *P* values (Table 2) from enrichment analysis and impact values from topology analysis, as shown in Fig. 5a, b. The topological score (*X*-axis) and the size of each circle represent the importance of different metabolites and their impact values in the enriched pathways, while the color of each circle based on the *P* value of the enrichment analysis (*Y*-axis) represents the statistical significance of the overall metabolic changes in the pathways. The pathway from yellow to red indicates a growing impact (Table 3). From this analysis, the citrate cycle (TCA cycle); fatty acid metabolism; glycolysis or gluconeogenesis; fatty acid elongation in mitochondria; alanine, aspartate, and glutamate metabolism; and glyoxylate and dicarboxylate metabolism were identified as the most perturbed metabolic pathways according to − log(*P* values) after si-Pellino1 treatment in the LPS-induced NRCMs. Protein interaction analysis found that multiple significantly enriched disturbed metabolic pathways were markedly related to the adipocytokine signaling pathway and PPAR signaling pathway (Fig. 6a, b).

**Table 2:** Impact analysis of metabolic set associations with Pellion1 treatment relative to the control

Pathway	Total	Expected	Hits	Raw *P*	− log(*P*)	Holm adjust	FDR	Impact
Citrate cycle (TCA cycle)	20	2.368	16	2.43E−12	26.743	1.97E−10	1.97E−10	0.73925
Fatty acid metabolism	39	4.6177	22	9.59E−12	25.37	7.67E−10	3.88E−10	0.75669
Glycolysis or gluconeogenesis	26	3.0785	17	9.92E−11	23.034	7.84E−09	2.68E−09	0.55585
Fatty acid elongation in mitochondria	27	3.1969	16	3.08E−09	19.599	2.40E−07	5.69E−08	0.79938
Alanine, aspartate, and glutamate metabolism	24	2.8417	15	3.51E−09	19.468	2.70E−07	5.69E−08	0.72784
Glyoxylate and dicarboxylate metabolism	16	1.8944	12	6.26E−09	18.889	4.76E−07	8.45E−08	1
Arginine and proline metabolism	44	5.2097	19	9.33E−08	16.188	6.99E−06	1.08E−06	0.63745
Pantothenate and CoA biosynthesis	15	1.776	10	7.45E−07	14.11	5.51E−05	7.54E−06	0.22449
Pyruvate metabolism	22	2.6049	12	1.16E−06	13.663	8.50E−05	1.05E−05	0.56143
Galactose metabolism	26	3.0785	13	1.50E−06	13.41	0.00010803	1.10E−05	0.32711
Glutathione metabolism	26	3.0785	13	1.50E−06	13.41	0.00010803	1.10E−05	0.68891
Beta-alanine metabolism	19	2.2496	10	1.49E−05	11.113	0.0010442	0.00010069	0.48148
Glycine, serine, and threonine metabolism	32	3.7889	13	2.62E−05	10.549	0.0018097	0.00015509	0.20398
Butanoate metabolism	20	2.368	10	2.68E−05	10.527	0.0018228	0.00015509	0.63768
Pentose and glucuronate interconversions	14	1.6576	8	5.28E−05	9.849	0.0035374	0.00028511	0.45455
Tryptophan metabolism	41	4.8545	13	0.00050418	7.5926	0.033276	0.0025524	0.44438
Starch and sucrose metabolism	23	2.7233	9	0.00069219	7.2757	0.044992	0.0031568	0.71075
Valine, leucine, and isoleucine biosynthesis	11	1.3024	6	0.00070151	7.2623	0.044992	0.0031568	0.99999
Histidine metabolism	15	1.776	7	0.00081193	7.1161	0.051152	0.0034614	0.30108
Valine, leucine, and isoleucine degradation	38	4.4993	12	0.00087151	7.0453	0.054034	0.0035296	0.18843
Propanoate metabolism	20	2.368	8	0.0011771	6.7447	0.071801	0.0045401	0.22414
Glycerophospholipid metabolism	30	3.5521	10	0.001499	6.5029	0.089942	0.0055191	0.37407
Aminoacyl-tRNA biosynthesis	67	7.933	16	0.0035245	5.648	0.20795	0.012412	0.03448
Synthesis and degradation of ketone bodies	5	0.59201	3	0.013613	4.2968	0.78953	0.045942	0.4
One carbon pool by folate	9	1.0656	4	0.014801	4.2131	0.84364	0.047954	0.74699
Pentose phosphate pathway	19	2.2496	6	0.018266	4.0027	1	0.056904	0.49197
Tyrosine metabolism	42	4.9729	9	0.051501	2.9662	1	0.1545	0.45681
Nitrogen metabolism	9	1.0656	3	0.079962	2.5262	1	0.22334	0
Ascorbate and aldarate metabolism	9	1.0656	3	0.079962	2.5262	1	0.22334	0.8
Cysteine and methionine metabolism	28	3.3153	6	0.10361	2.2672	1	0.27974	0.42481
d-glutamine and d-glutamate metabolism	5	0.59201	2	0.10954	2.2114	1	0.28622	1
Amino sugar and nucleotide sugar metabolism	37	4.3809	7	0.13818	1.9792	1	0.34978	0.31198
Fructose and mannose metabolism	19	2.2496	4	0.1792	1.7192	1	0.43986	0.21072
Lysine degradation	20	2.368	4	0.20462	1.5866	1	0.48747	0.32608
Terpenoid backbone biosynthesis	15	1.776	3	0.25816	1.3542	1	0.59745	0.10753
Methane metabolism	9	1.0656	2	0.28941	1.2399	1	0.65117	0.4
Ubiquinone and other terpenoid-quinone biosynthesis	3	0.35521	1	0.31501	1.1552	1	0.68961	0
Phenylalanine, tyrosine, and tryptophan biosynthesis	4	0.47361	1	0.39629	0.92562	1	0.84471	0.5
Thiamine metabolism	7	0.82882	1	0.58694	0.53284	1	1	0
Inositol phosphate metabolism	26	3.0785	3	0.61111	0.49247	1	1	0.13525
Taurine and hypotaurine metabolism	8	0.94722	1	0.63609	0.45242	1	1	0
Glycerolipid metabolism	18	2.1312	2	0.6481	0.4337	1	1	0.13031
Phenylalanine metabolism	9	1.0656	1	0.67942	0.38651	1	1	0
Pyrimidine metabolism	41	4.8545	4	0.73526	0.30753	1	1	0.07706
Nicotinate and nicotinamide metabolism	13	1.5392	1	0.80714	0.21426	1	1	0
Glycosylphosphatidylinositol (GPI)-anchor biosynthesis	14	1.6576	1	0.83019	0.18611	1	1	0
Porphyrin and chlorophyll metabolism	27	3.1969	2	0.84865	0.16411	1	1	0.02205
Selenoamino acid metabolism	15	1.776	1	0.85049	0.16194	1	1	0
Folate biosynthesis	16	1.8944	1	0.86839	0.14112	1	1	0.06087
Sphingolipid metabolism	21	2.4864	1	0.93052	0.07201	1	1	0
Fatty acid biosynthesis	43	5.0913	2	0.97169	0.028721	1	1	0.08483
Biosynthesis of unsaturated fatty acids	42	4.9729	1	0.99538	0.004632	1	1	0

**Table 3:** Enrichment analysis of metabolic set associations with Pellino1 treatment relative to the control

Pathway	Total	Expected	Hits	Raw *P*	Holm *P*	FDR
Fatty acid metabolism	43	6.97	22	5.58E−08	5.47E−06	5.47E−06
Fatty acid elongation in mitochondria	35	5.67	19	1.44E−07	1.40E−05	7.07E−06
Warburg effect	58	9.4	23	8.32E−06	0.000799	0.000272
Glycine and serine metabolism	59	9.56	22	4.28E−05	0.00406	0.00105
Glutamate metabolism	49	7.94	18	0.000286	0.0268	0.0056
Aspartate metabolism	35	5.67	14	0.000519	0.0483	0.00727
Gluconeogenesis	35	5.67	14	0.000519	0.0483	0.00727
Glutathione metabolism	21	3.4	10	0.000672	0.0611	0.00823
Glycolysis	25	4.05	11	0.000826	0.0743	0.00853
Arginine and proline metabolism	53	8.59	18	0.00087	0.0774	0.00853
Butyrate metabolism	19	3.08	9	0.00133	0.117	0.0119
Tryptophan metabolism	60	9.73	19	0.00165	0.143	0.0132
Mitochondrial beta-oxidation of short-chain saturated fatty acids	27	4.38	11	0.00179	0.154	0.0132
Glucose-alanine cycle	13	2.11	7	0.00188	0.16	0.0132
Citric acid cycle	32	5.19	12	0.00258	0.216	0.0168
Urea cycle	29	4.7	11	0.00352	0.292	0.0216
Galactose metabolism	38	6.16	13	0.00439	0.36	0.0239
Transfer of acetyl groups into mitochondria	22	3.57	9	0.00461	0.373	0.0239
Beta-alanine metabolism	34	5.51	12	0.00463	0.373	0.0239
Pyruvate metabolism	48	7.78	15	0.00598	0.472	0.0293
Mitochondrial beta-oxidation of medium-chain saturated fatty acids	27	4.38	10	0.00657	0.512	0.0306
Ketone body metabolism	13	2.11	6	0.0106	0.813	0.0471
Propanoate metabolism	42	6.81	13	0.0113	0.862	0.0483
Lysine degradation	30	4.86	10	0.015	1	0.0612
Valine, leucine, and isoleucine degradation	60	9.73	16	0.0232	1	0.0908
Methionine metabolism	43	6.97	12	0.0337	1	0.127
Spermidine and spermine biosynthesis	18	2.92	6	0.0566	1	0.205
Ammonia recycling	32	5.19	9	0.06	1	0.21
Malate–aspartate shuttle	10	1.62	4	0.063	1	0.213
Mitochondrial beta-oxidation of long-chain saturated fatty acids	28	4.54	8	0.0686	1	0.224
Ethanol degradation	19	3.08	6	0.0719	1	0.224
Histidine metabolism	43	6.97	11	0.0733	1	0.224
Pentose phosphate pathway	29	4.7	8	0.0822	1	0.237
Folate metabolism	29	4.7	8	0.0822	1	0.237
Catecholamine biosynthesis	20	3.24	6	0.0895	1	0.251
Tyrosine metabolism	72	11.7	16	0.105	1	0.285
Pantothenate and CoA biosynthesis	21	3.4	6	0.109	1	0.289
Starch and sucrose metabolism	31	5.03	8	0.114	1	0.293
Methylhistidine metabolism	4	0.648	2	0.125	1	0.309
Alanine metabolism	17	2.76	5	0.126	1	0.309
Lactose degradation	9	1.46	3	0.167	1	0.399
Phospholipid biosynthesis	29	4.7	7	0.176	1	0.411
Mitochondrial electron transport chain	19	3.08	5	0.181	1	0.413
Glycerolipid metabolism	25	4.05	6	0.207	1	0.46
Nucleotide sugar metabolism	20	3.24	5	0.212	1	0.462
Phytanic acid peroxisomal oxidation	26	4.21	6	0.235	1	0.5
Betaine metabolism	21	3.4	5	0.244	1	0.509
Glycerol phosphate shuttle	11	1.78	3	0.258	1	0.516
Trehalose degradation	11	1.78	3	0.258	1	0.516
Amino sugar metabolism	33	5.35	7	0.279	1	0.546
Phenylalanine and tyrosine metabolism	28	4.54	6	0.294	1	0.566
Vitamin K metabolism	14	2.27	3	0.402	1	0.743
Phosphatidylcholine biosynthesis	14	2.27	3	0.402	1	0.743
Cysteine metabolism	26	4.21	5	0.417	1	0.748
Fructose and mannose degradation	32	5.19	6	0.42	1	0.748
Homocysteine degradation	9	1.46	2	0.442	1	0.761
De novo triacylglycerol biosynthesis	9	1.46	2	0.442	1	0.761
Pyruvaldehyde degradation	10	1.62	2	0.5	1	0.845
Beta oxidation of very-long-chain fatty acids	17	2.76	3	0.537	1	0.888
Cardiolipin biosynthesis	11	1.78	2	0.554	1	0.888
Androstenedione metabolism	24	3.89	4	0.563	1	0.888
Estrone metabolism	24	3.89	4	0.563	1	0.888
Retinol metabolism	37	6	6	0.571	1	0.888
Oxidation of branched chain fatty acids	26	4.21	4	0.63	1	0.929
Plasmalogen synthesis	26	4.21	4	0.63	1	0.929
Sphingolipid metabolism	40	6.48	6	0.652	1	0.929
Ubiquinone biosynthesis	20	3.24	3	0.654	1	0.929
Lactose synthesis	20	3.24	3	0.654	1	0.929
Threonine and 2-oxobutanoate degradation	20	3.24	3	0.654	1	0.929
Steroid biosynthesis	48	7.78	7	0.685	1	0.959
Pterine biosynthesis	29	4.7	4	0.718	1	0.979
Carnitine synthesis	22	3.57	3	0.719	1	0.979
Phenylacetate metabolism	9	1.46	1	0.798	1	1
Androgen and estrogen metabolism	33	5.35	4	0.81	1	1
Fatty acid biosynthesis	35	5.67	4	0.846	1	1
d-arginine and d-ornithine metabolism	11	1.78	1	0.859	1	1
Degradation of superoxides	11	1.78	1	0.859	1	1
Pyrimidine metabolism	59	9.56	7	0.87	1	1
Nicotinate and nicotinamide metabolism	37	6	4	0.876	1	1
Taurine and hypotaurine metabolism	12	1.95	1	0.882	1	1
Phosphatidylethanolamine biosynthesis	12	1.95	1	0.882	1	1
Thyroid hormone synthesis	13	2.11	1	0.901	1	1
Caffeine metabolism	24	3.89	2	0.922	1	1
Inositol metabolism	33	5.35	3	0.924	1	1
Inositol phosphate metabolism	26	4.21	2	0.942	1	1
Phosphatidylinositol phosphate metabolism	17	2.76	1	0.952	1	1
Porphyrin metabolism	40	6.48	3	0.97	1	1
Purine metabolism	74	12	7	0.971	1	1
Arachidonic acid metabolism	69	11.2	5	0.993	1	1
Selenoamino acid metabolism	28	4.54	1	0.993	1	1
Steroidogenesis	43	6.97	2	0.996	1	1
Bile acid biosynthesis	65	10.5	3	0.999	1	1

**Fig. 7:**
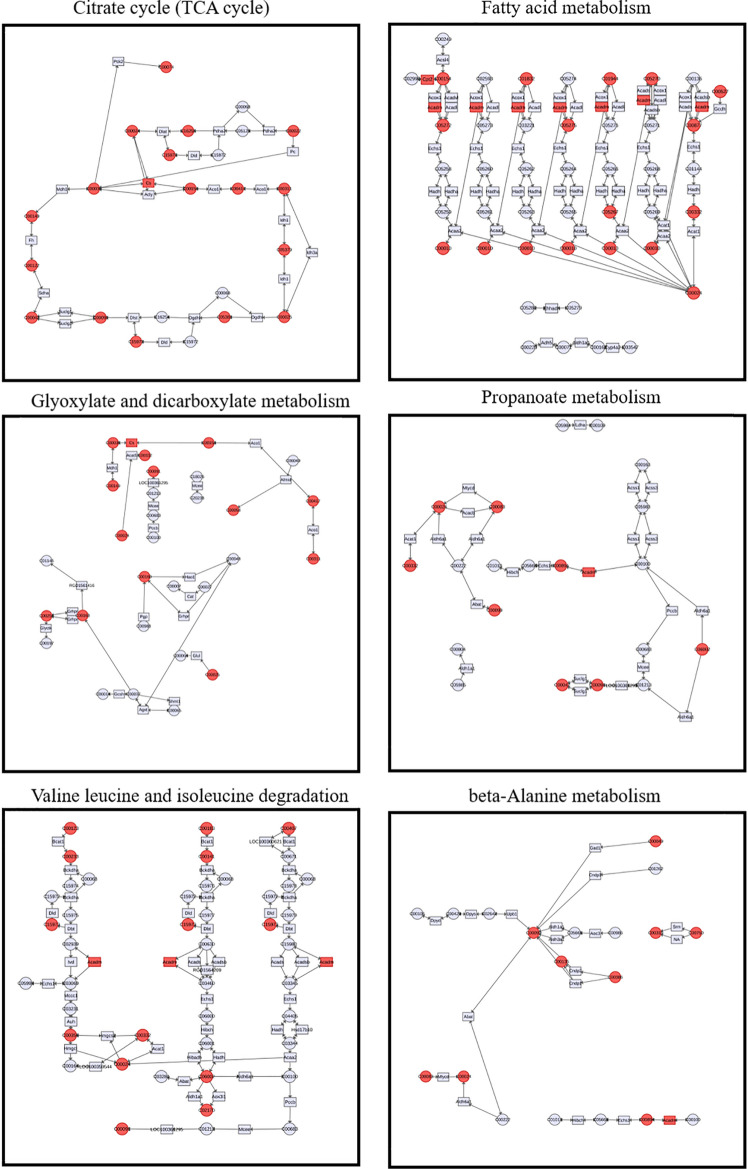
Interaction analysis of gene and metabolite associations with si-Pellino1 treatment. Correlation networks of the citrate cycle (TCA cycle); fatty acid metabolism; glyoxylate and dicarboxylate metabolism; propanoate metabolism; valine, leucine, and isoleucine degradation and beta-alanine metabolism showed the inner relationships between metabolites and genes that we previously explored in the main enriched metabolic pathways showing changes

To investigate the relationships between metabolites and genes that we previously explored in the main enriched metabolic pathways showing alterations, we generated specific correlation networks of the citrate cycle (TCA cycle), fatty acid metabolism, glyoxylate, and dicarboxylate metabolism and propanoate metabolism, as well as valine leucine and isoleucine degradation and beta-alanine metabolism, as shown in Fig. 7. The results shown above verified that si-Pellino1 treatment could alleviate or even reverse the LPS-induced cellular damage by altering cardiac fuel and energy metabolism accompanied by changes in key genes (Cs, Cpt2, and Acadm) and metabolites (3-oxoocotanoyl-CoA, hydroxypyruvic acid, lauroyl-CoA, and NADPH).

## Discussion

Bacterial myocarditis is a common clinical sepsis syndrome caused mainly by inflammation, which can contribute to adverse cardiovascular outcomes, end-organ dysfunction, and even mortality (Li and Sun 2007; Schauvliege et al. 2007).

LPS-trigged excessive host responses have been established to result in systemic inflammatory conditions, including myocarditis and sepsis, mainly by activating the Toll-like receptor (TLR) family, the major pattern-recognition receptors (PRRs) family of host cells (O’Neill et al. 2009; Akira et al. 2006), which fuels our interest in exploring the signaling function of TLRs after LPS stimulation. Previous studies have shown that TLRs-activated signal transduction pathways which determine the vital role of TLRs in inflammatory disorders depend on the binding between their intracellular domain (called TIR domain) and intracellular adaptors, including myeloid differentiation factor 88 (MyD88), Mal (TIRAP), TRIF (TICAM-1), and TRAM (TICAM-2) (Kawai and Akira 2007; Dunne and O’Neill 2003). Upon LPS stimulation, the phosphorylation cascade of MyD88 initiated by TLRs mediates the recruitment and activation of IL-1R-associated kinases (IRAKs), further triggering the activation of the nuclear factor-κB (NF-κB) family, resulting in the production of pro-inflammatory mediators helping to shape the immune response and eradicate infection (Janssens and Beyaert 2003).

As an intrinsic ubiquitin E3 ligase responsible for receptor-interacting protein 1 (RIP1) ubiquitination in the TLR/IL-1R signaling, Pellino is an evolutionarily conserved scaffold protein that could catalyze the polyubiquitylation of key molecule involved in the tumor necrosis factor receptor-associated factor (TRAF)-dependent TLR signaling pathway by interacting with its highly homologous family, IRAKs, in an MyD88-dependent manner after stimulation in mammals (Grosshans et al. 1999a; Schauvliege et al. 2007).

Pellino1, a mammalian counterpart of Pellino, have been recognized to be highly required for maintaining TLR-mediated NF-κB nuclear translocation and binding activity due to its value in forming Pellino1–IRAK1–IRAK4–TRAF6 signaling complex after the induction of inflammatory response (Moynagh 2009; Jiang et al. 2003), indicating the causal relationship between Pellino1 and TLR-mediated inflammatory response. In addition to TRAF6, a recent study found that the activation of TIR-domain-containing adapter-inducing interferon-β (TRIF)-dependent signaling induced-by Pellino1-mediated ubiquitination of RIP1 also contributed to the upregulation of NF-κB (Sato et al. 2003; Chang et al. 2009). Moreover, Pellino1 deficiency may interfere with the ubiquitination of RIP1 and TRAF6 which are needed for TLR3/4-mediated pro-inflammatory gene induction, thereby increasing the resistance of mice to LPS-induced lethality (Chang et al. 2009; Skaug et al. 2009). Collectively, the importance of Pellino1 in LPS-induced inflammatory signaling makes it reasonable to assume that suppressing Pellino1 do be especially effective in attenuating LPS-induced myocarditis.

As the unifying link between heart and cardiac function, changed cardiac metabolism has been evidenced to be causative in cardiomyocytes dysfunction induced by various physiological and pathological causes, including exercise and pressure overload (Gibb and Hill 2018). During infections, cell-intrinsic metabolism is no longer considered as a physical process that meets the basic demand of energy for heart contraction (Newsholme et al. 1986). In addition to supporting tissue homeostasis, pathogen-activated reprogramming of metabolic pathways also contributes to the production of metabolic intermediates and end products (Williams and O’Neill 2018). The immune-metabolic framework consisted of kinds of regulatory metabolites that play a vital role in regulating cardiac function, and determining the fate of an infection (Chauhan and Saha 2018).

Recent advances in LC–MS/MS-based omics uncovered that changed metabolites under pathological states could lead to the progression of inflammation (Ishihara et al. 2019). It has been shown that myeloperoxidase (MPO) could mediate primarily host defense reactions by regulating peroxidation of polyunsaturated fatty acids which is the characteristic feature of inflammation during acute inflammation (Winterbourn et al. 2000; Kubala et al. 2010). The withdrawal of the synthesis of bioactive metabolites of arachidonic acid (AA) and linoleic acid (LA) metabolism may ameliorate the severity of sepsis by modifying inflammatory-related cellular signaling pathways (Cook 2005; Nauseef 2001). Besides, the l-Tryptophan (Trp)–kynurenine (Kyn) pathway, which is essential for protein synthesis, was reported to improve acute viral myocarditis (Hoshi et al. 2012).

Metabolic remodeling after LPS stimulation may modify the downstream genes and proteins by triggering the mammalian target of rapamycin (mTOR), AMP-activated protein kinase (AMPK), and nuclear factor-κB (NF-κB) signaling pathways involved in many pathological conditions, such as atherosclerosis, indicating that it is necessary to emphasize the importance of metabolites in LPS-induced pro-inflammatory response (Byles et al. 2013; Sag et al. 2008; Wu et al. 2020). As the main source of energy for cardiac mechanical work, fatty acid (FA) can be transported by fatty acid translocase (FAT/CD36) and other transport proteins from the cytosol to the mitochondria and then form acyl-CoA to enter β-oxidation (van der Vusse et al. 2000; Berndt et al. 2019; Girones et al. 2014). The peroxisome proliferator-activated receptor subfamily is vital in the epigenetic regulation of FA utilization. It has been reported that the main three members of the PPAR nuclear receptor subfamily, PPARα, PPARδ, and PPARγ, are all involved in synergistic modulation of cardiomyocytes energy metabolism (Lopaschuk et al. 2010). Furthermore, in in vitro models of mice, the activation of PPARα has been identified to increase the expression of downstream genes, including CD36, MCPT1, and MCAD, which play different important roles in cardiac FA utilization (Lopaschuk et al. 2010; Schoonjans et al. 1996; Mistry and Cresci 2010; Huss and Kelly 2004). The induced transcriptional activities of both PPARs and ERRs could be coactivated by binding to PGC-1α (Huss et al. 2004; Schreiber et al. 2004). Recently, the PGC-1α/PPARα and PGC-1α/ERRα pathways are involved in regulating cardiac mitochondrial fatty acid oxidation genes (Lehman et al. 2000).

PGC-1α, an important regulator of mitochondrial activity and fatty acid oxidative metabolism, is enriched in the heart, which has high oxidative metabolic rates (Lehman et al. 2000; Lin et al. 2002; Knutti and Kralli 2001). Compared to those of PGC-1β, which is also confirmed to be necessary for aerobic mitochondrial respiration, post-translational modifications of PGC-1α bound with transcriptional partners could better control complex cardiac cellular energy metabolism by activating downstream endogenous gene promoters, including MCAD (St-Pierre et al. 2003; Rowe et al. 2010; Huss et al. 2002). Interestingly, in our study, silencing Pellino1 reversed the LPS-induced decrease in the expression of transcription factors and regulators in NRCMs, showing that Pellino1 plays a role in regulating fatty acid metabolism.

After analyzing the metabolome data, we found 169 unique metabolites that differed significantly in si-Pellino1 and LPS-treated, LPS-treated, and untreated control NRCMs. Then, the TCA cycle, fatty acid metabolism, and glycolysis or gluconeogenesis, along with fatty acid elongation in mitochondria and alanine, aspartate and glutamate metabolism, as well as glyoxylate and dicarboxylate metabolism, were identified as the most perturbed metabolic pathways according to *P* values from enrichment analysis and impact values from topology analysis.

Moreover, lipopolysaccharide has been reported to regulate the myocardial expression of the FAO enzyme and oxidation rates by deactivating the kinetic response of the PGC-1 axis, which is associated with systemic inflammation and sepsis-induced by LPS stimulation (Beutler and Rietschel 2003; Rossi et al. 2007; Puigserver et al. 2001; Feingold et al. 2004). Our results found that LPS altered cardiac fuel and energy metabolism accompanied by changes in key genes (Cs, Cpt2, and Acadm) and metabolites (3-oxoocotanol-CoA, hydroxypyruvic acid, lauroyl-CoA, and NADPH) in NRCMs. Furthermore, for the first time, we confirmed that Pellino1, which has been identified to activate NF-κB and TLR signaling pathways by mediating proinflammatory genes in innate immunity (Chang et al. 2009; Kawai and Akira 2007; Choi et al. 2006; Vallabhapurapu and Karin 2009), could partly reverse or restore the disturbed cardiomyocytes energy metabolism through mechanisms involving glycolysis, lipid metabolism, citrate cycle, and some other types of metabolism after LPS stimulation.

Glucose utilization is also highly linked to various physiological processes, including cardiomyocytes energy metabolism, which can affect mitochondrial function by coregulation with FA utilization (Randle 1998). GLUT1 and GLUT4, the common and major glucose transporters of glycogen-derived glucose in the heart, play a basic and important role in basal myocardial glucose uptake, suggesting that the decreased expression of GLUT4 may be harmful to cardiac glucose metabolism (Henning et al. 1996; Aerni-Flessner et al. 2012). It has been reported that pathological cardiac hypertrophy could accelerate glycolysis (Allard et al. 1994; Cheng et al. 2017).

In our study, si-Pellino1 alleviated the LPS-induced decrease in GLUT4 expression in NRCMs. The observed changes in two pivotal intermediates, succinic acid, and malic acid, were partly reversed after si-Pellino1 treatment, suggesting that silencing Pellino1 in LPS-induced myocarditis improved LPS-induced glucose metabolic dysfunction.

The metabolic profiles analyzed in our study could partly explain the therapeutic value of silencing Pellino1 in LPS-induced myocarditis. However, this report has some limitations that need to be considered. In our study, the samples were extracted from NRCMs cultivated in vitro. As a result, given the different metabolic environments, we lack experimental data from in vivo studies. Additionally, targeted metabolomics is an optimal method to identify the differences in metabolites. Consequently, these issues remain to be examined in the future.

Thus, the LC–MS/MS metabonomic method shed new light on the mechanism of the pathological development of bacterial myocarditis and was also used to assess the efficacy of si-Pellino1 treatment. Importantly, the altered metabolites associated with the TCA cycle, fatty acid metabolism, and glycolytic metabolism may be used as potential specific biomarkers corresponding to LPS-induced myocarditis. Moreover, combined with restoration of the abnormal metabolism and disturbed expression of transcription factors involved in cardiac fuel and energy metabolism, silencing Pellino1 showed cardioprotective value, revealing that silencing Pellino1 could be a promising therapeutic strategy against LPS-induced myocarditis or other pathologies in future clinical trials. In addition, considering the pro-inflammatory role of Pellino1 reported in the sepsis-induced lung injury model and COPD-induced inflammatory airway responses, silencing Pellino1 can also ameliorate acute or chronic lung pneumonia (Liu et al. 2021; Marsh et al. 2020; Hughes et al. 2019). Other than the heart and lung, the regulatory effects of Pellino1 on TLR/IL-1R signaling make it reasonable to assume that silencing Pellino1 may be targeted therapeutically in different inflammatory disorders associated with TLR/IL-1R signaling, which remains to be further explored in the future.

## Supplementary Information

Below is the link to the electronic supplementary material.

Supplementary file1 (DOCX 61 KB)

Supplementary file2 All samples induced by LPS or Pellino1-silencing adenovirus were analyzed and evaluated by LC–MS. 4A: Total ion chromatography (TIC) of the analyzed QC samples was compared with the spectral overlap by means of QC sample spectrogram comparison. x-axis: intensity; y-axis: intension time/minutes. 4B-E: Normalization of the sample. The samples were normalized by median sample (TIF 17783 KB)

Supplementary file3 Metabonimics data were normalized by date scaling. A-D: Normalization of the data. The ion intensity of each metabolite was normalized with mean of metabolite (TIF 15649 KB)
