# Supplementary material for: Pellino1 deficiency reprograms cardiomyocytes energy metabolism in lipopolysaccharide-induced myocardial dysfunction
Source: Amino Acids. 2021 Apr 22;53(5):713–37. doi: 10.1007/s00726-021-02978-w (PMC8128834; doi:10.1007/s00726-021-02978-w)
Supplement: Supplementary file 1 — Supplementary file1 (DOCX 61 KB) [file 726_2021_2978_MOESM1_ESM.docx]

Table S1: Sequence for qRT-PCR.
