# Supplementary figures and images for: Pellino1 deficiency reprograms cardiomyocytes energy metabolism in lipopolysaccharide-induced myocardial dysfunction
Source: Amino Acids. 2021 Apr 22;53(5):713–37. doi: 10.1007/s00726-021-02978-w (PMC8128834; doi:10.1007/s00726-021-02978-w)

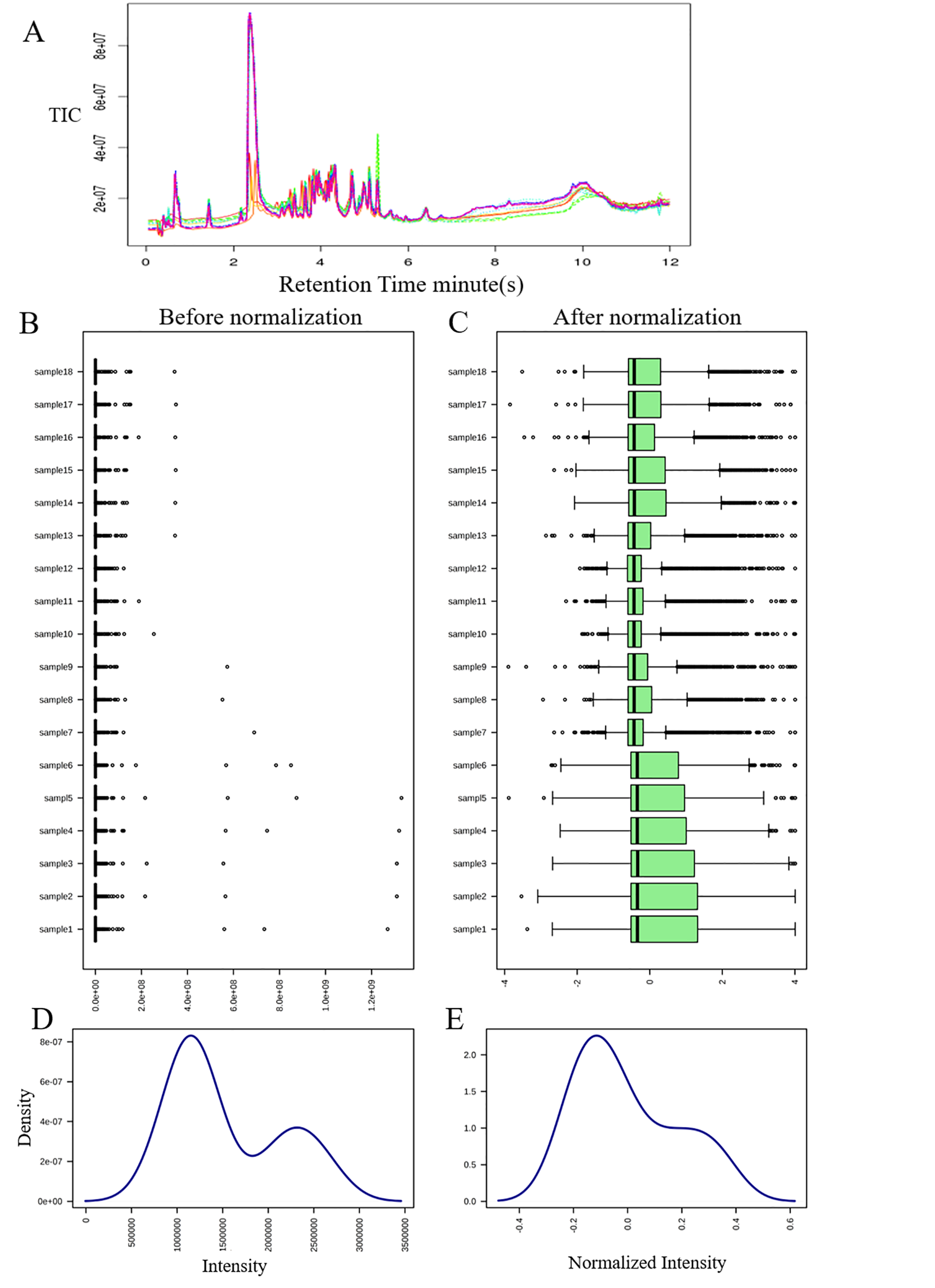

Supplement: Supplementary file 2 — Supplementary file2 All samples induced by LPS or Pellino1-silencing adenovirus were analyzed and evaluated by LC–MS. 4A: Total ion chromatography (TIC) of the analyzed QC samples was compared with the spectral overlap by means of QC sample spectrogram comparison. x-axis: intensity; y-axis: intension time/minutes. 4B-E: Normalization of the sample. The samples were normalized by median sample (TIF 17783 KB) [file 726_2021_2978_MOESM2_ESM.tif]

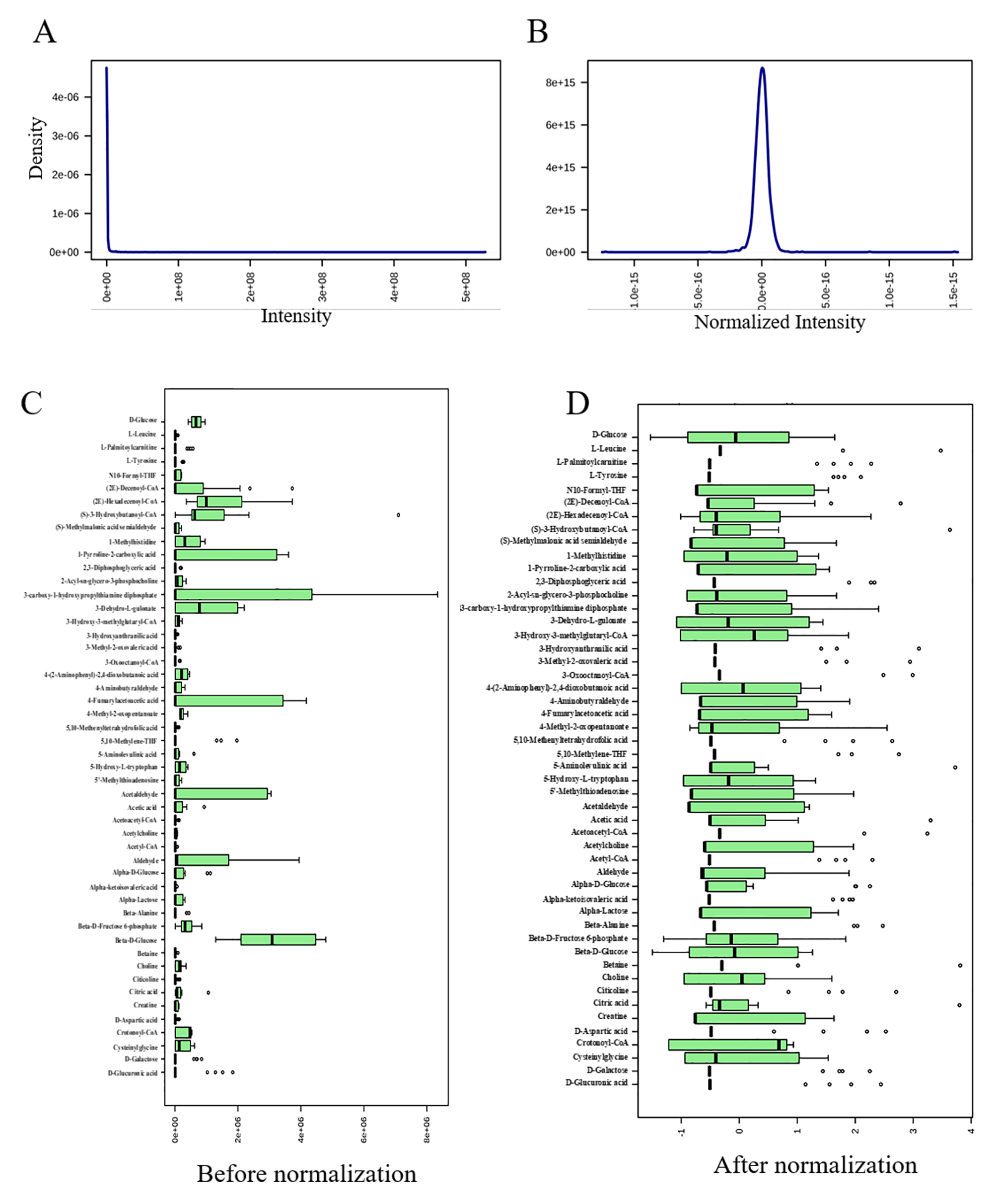

Supplement: Supplementary file 3 — Supplementary file3 Metabonimics data were normalized by date scaling. A-D: Normalization of the data. The ion intensity of each metabolite was normalized with mean of metabolite (TIF 15649 KB) [file 726_2021_2978_MOESM3_ESM.tif]
